# Superdiffusive limits for deterministic fast–slow dynamical systems

**DOI:** 10.1007/s00440-020-00988-5

**Published:** 2020-07-16

**Authors:** Ilya Chevyrev, Peter K. Friz, Alexey Korepanov, Ian Melbourne

**Affiliations:** 1 School of Mathematics, University of Edinburgh, Edinburgh, EH9 3FD UK; 2 Institut für Mathematik, Technische Universität Berlin, Berlin, Germany; 3 Weierstraß-Institut für Angewandte Analysis und Stochastik, Berlin, Germany; 4 Department of Mathematics, University of Exeter, Exeter, EX4 4QF UK; 5 Mathematics Institute, University of Warwick, Coventry, CV4 7AL UK

**Keywords:** Primary 37A50, Secondary 60L20

## Abstract

We consider deterministic fast–slow dynamical systems on $\mathbb {R}^m\times Y$ of the form

$$\begin{aligned} {\left\{ \begin{array}{ll} x_{k+1}^{(n)} = x_k^{(n)} + n^{-1} a\big (x_k^{(n)}\big ) + n^{-1/\alpha } b\big (x_k^{(n)}\big ) v(y_k), \\ y_{k+1} = f(y_k), \end{array}\right. } \end{aligned}$$

where $\alpha \in (1,2)$. Under certain assumptions we prove convergence of the *m*-dimensional process $X_n(t)= x_{\lfloor nt \rfloor }^{(n)}$ to the solution of the stochastic differential equation

$$\begin{aligned} \mathrm {d} X = a(X)\mathrm {d} t + b(X) \diamond \mathrm {d} L_\alpha , \end{aligned}$$

where $L_\alpha$ is an $\alpha$-stable Lévy process and $\diamond$ indicates that the stochastic integral is in the Marcus sense. In addition, we show that our assumptions are satisfied for intermittent maps *f* of Pomeau–Manneville type.

## Introduction

Averaging and homogenisation for systems with multiple timescales is a longstanding and very active area of research [34]. We focus particularly on homogenisation, where the limiting equation is a stochastic differential equation (SDE). Recently there has been considerable interest in the case where the underlying multiscale system is deterministic, see [9–11, 16, 20, 21, 24, 32, 35] as well as our survey paper [8]. Almost all of this previous research has been concerned with the case where the limiting SDE is driven by Brownian motion. Here, we consider the case where the limiting SDE is driven by a superdiffusive $\alpha$-stable Lévy process.

Let $\alpha \in (1,2)$. The multiscale equations that we are interested in have the form

1.1 $$\begin{aligned} {\left\{ \begin{array}{ll} x_{k+1}^{(n)} = x_k^{(n)} + n^{-1} a\big (x_k^{(n)}\big ) + n^{-1/\alpha } b\big (x_k^{(n)}\big ) v(y_k) , \\ y_{k+1} = f(y_k) \end{array}\right. } \end{aligned}$$

defined on ${\mathbb {R}}^m\times Y$ where *Y* is a bounded metric space. Here

$$\begin{aligned} a :{\mathbb {R}}^m\rightarrow {\mathbb {R}}^m , \quad b :{\mathbb {R}}^m\rightarrow {\mathbb {R}}^{m\times d} , \quad v :Y\rightarrow {\mathbb {R}}^d , \quad f:Y\rightarrow Y . \end{aligned}$$

It is assumed that the fast dynamical system $f:Y\rightarrow Y$ has an ergodic invariant probability measure $\mu$ and exhibits superdiffusive behaviour; specific examples for such *f* are described below. Let $v :Y\rightarrow {\mathbb {R}}^d$ be Hölder with $\int v\mathrm {d}\mu =0$. Define for $n\ge 1$,

1.2 $$\begin{aligned} W_n(t)=n^{-1/\alpha }\sum _{j=0}^{\lfloor nt \rfloor -1}v\circ f^j . \end{aligned}$$

Then $W_n$ belongs to $D([0,1], {\mathbb {R}}^d)$, the Skorokhod space of càdlàg functions, and can be viewed as a random process on the probability space $(Y, \mu )$ depending on the initial condition $y_0\in Y$. As $n \rightarrow \infty$, the sequence of random variables $W_n(1)$ converges weakly in ${\mathbb {R}}^d$ to an $\alpha$-stable law, and the process $W_n$ converges weakly in $D([0,1],{\mathbb {R}}^d)$ to the corresponding $\alpha$-stable Lévy process $L_\alpha$.

Now consider $x_0^{(n)}=\xi _n \in {\mathbb {R}}^m$, and solve (1.1) to obtain $(x_k^{(n)},y_k)_{k\ge 0}$ depending on the initial condition $y_0\in (Y,\mu )$. Define the càdlàg process $X_n\in D([0,1],{\mathbb {R}}^m)$ given by $X_n(t) = x_{\lfloor nt \rfloor }^{(n)}$; again we view this as a process on $(Y,\mu )$. Our aim is to show, under mild regularity assumptions on the functions $a:{\mathbb {R}}^m\rightarrow {\mathbb {R}}^m$ and $b :{\mathbb {R}}^m \rightarrow {\mathbb {R}}^{m \times d}$, that $X_n\rightarrow _w X$ where *X* is the solution of the SDE

1.3 $$\begin{aligned} \mathrm {d}X = a(X)\mathrm {d}t + b(X) \diamond \mathrm {d}L_\alpha , \qquad X(0) = \xi \end{aligned}$$

and $\xi = \lim _{n\rightarrow \infty } \xi _n$. Here, $\diamond$ indicates that the SDE is in the Marcus sense [29] (see [2, 5, 25] for the general theory of Marcus SDEs and their applications).

Previously such a result was shown by Gottwald and Melbourne [16, Section 5] in the special case $d=m=1$. Generally the method in [16] works provided the noise is exact, that is $d=m$ and $b=(Dr)^{-1}$ for some diffeomorphism $r:{\mathbb {R}}^m\rightarrow {\mathbb {R}}^m$, but cannot handle the general situation considered here where the noise term is typically not exact. There are three main complications:

1. In the case of exact noise, it is possible to reduce to the case $b \equiv {\mathrm {id}}$ by a change of coordinates, similar to Wong–Zakai [45]. The general situation necessitates the use of alternative tools such as rough paths. In particular, weak convergence of $W_n$ is no longer sufficient and we require in addition that $W_n$ is tight in *p*-variation. This is shown in Theorem 1.3 below for specific examples, and in Sect. 6 for a large class of deterministic dynamical systems $f:Y\rightarrow Y$.
2. Since the results for exact noise are achieved by a change of coordinates, the sense of convergence for $W_n$ is inherited by $X_n$. However, in general, even if $W_n\rightarrow _w L_\alpha$ in one of the standard Skorokhod topologies [40], this need not be the case for $X_n$. This phenomenon already appears in the simplest situations, as illustrated in Example 1.4. Hence we have to consider convergence of $X_n$ in generalised Skorokhod topologies as introduced recently in Chevyrev and Friz [7].
3. Rigorous results on convergence to *d*-dimensional stable Lévy processes in deterministic dynamical systems are only available for $d=1$, see [1, 22, 33, 42]. Hence one of the aims of this paper is to extend the dynamical systems theory to cover the case $d\ge 2$. See Theorem 1.1 below for instances of this, and Sect. 6 for a general treatment.

In the remainder of the introduction, we discuss some of the issues associated to these three complications. We also mention some examples of fast dynamical systems that lead to superdiffusive behaviour. The archetypal such dynamical systems are the intermittent maps introduced by Pomeau and Manneville [37]. Perhaps the simplest example [27] is the map $f:Y\rightarrow Y$, $Y=[0,1]$, with a neutral fixed point at 0:

1.4 $$\begin{aligned} f(y) = {\left\{ \begin{array}{ll} y (1 + 2^{1/\alpha } y^{1/\alpha }), &{}\quad y \in [0, \frac{1}{2}) , \\ 2y - 1, &{} \quad y \in [\frac{1}{2},1] . \end{array}\right. } \end{aligned}$$

**Fig. 1:**
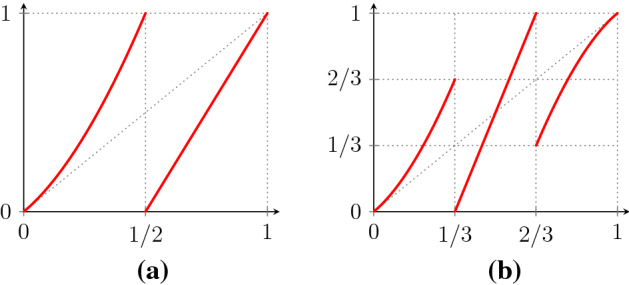
Examples of intermittent maps: **a** the map (1.4), **b** the map (1.5)

See Fig. 1a. Here, $\alpha >0$ is a real parameter and there is a unique absolutely continuous invariant probability measure $\mu$ for $\alpha >1$. Let $v:Y\rightarrow {\mathbb {R}}$ be Hölder with $\int _Y v\mathrm {d}\mu =0$ and $v(0)\ne 0$, and define $W_n$ as in (1.2). For $\alpha \in (1,2)$ it was shown by Gouëzel [17] (see also [46]) that $W_n(1)$ converges in distribution to an $\alpha$-stable law. By Melbourne and Zweimüller [33], the process $W_n$ converges weakly to the corresponding Lévy process $L_\alpha$ in the ${\mathcal {M}}_1$ Skorokhod topology on $D([0,1],{\mathbb {R}})$.

Now let $d\ge 2$. There are two versions of the ${\mathcal {M}}_1$ topology on $D([0,1],{\mathbb {R}}^d)$, see [43, Chapter 3.3]. In this paper we use the strong topology ${\mathcal {SM}}_1$. For $v:Y\rightarrow {\mathbb {R}}^d$ Hölder with $\int _Y v\mathrm {d}\mu =0$ and $v(0)\ne 0$, we prove convergence of $W_n$ to a *d*-dimensional Lévy process $L_\alpha$ in the ${\mathcal {SM}}_1$ topology.

The example (1.4) is somewhat oversimplified for our purposes since $L_\alpha$ is essentially one-dimensional, being supported on the line $\{c v(0): c \in {\mathbb {R}}\}$. This structure can be exploited in proving that $W_n\rightarrow _w L_\alpha$, though it is not clear if this simplifies the homogenisation result $X_n\rightarrow _w X$. To illustrate that we do not rely on one-dimensionality of the limiting process in any way, we consider an example with two neutral fixed points. (It is straightforward to extend to maps with a larger number of neutral fixed points.) Accordingly, our main example is the intermittent map $f :Y \rightarrow Y$, $Y= [0,1]$, with two symmetric neutral fixed points at 0 and 1:

1.5 $$\begin{aligned} f(y) = {\left\{ \begin{array}{ll} y\big (1+3^{1/\alpha } y^{1/\alpha }\big ), &{}\quad y \in [0, \frac{1}{3}) , \\ 3y-1, &{}\quad y\in [\frac{1}{3} , \frac{2}{3}) , \\ 1 - (1-y) \big (1 + 3^{1/\alpha } (1-y)^{1/\alpha }\big ), &{}\quad y\in [ \frac{2}{3},1] . \end{array}\right. } \end{aligned}$$

See Fig. 1b. Again $\alpha >0$ is a real parameter, there is a unique absolutely continuous invariant probability measure $\mu$ for $\alpha >1$, and we restrict to the range $\alpha \in (1,2)$.

As part of a result for a general class of nonuniformly expanding maps (Sect. 6) we prove:

### Theorem 1.1

Consider the intermittent map (1.4) or (1.5) with $\alpha \in (1,2)$ and let $v:Y\rightarrow {\mathbb {R}}^d$ be Hölder with $\int _Y v\mathrm {d}\mu =0$ and $v(0)\ne 0$, also $v(1)\ne 0$ in case of (1.5). Let ${{\,\mathrm{\mathbb {P}}\,}}$ be any probability measure on *Y* that is absolutely continuous with respect to Lebesgue, and regard $W_n$ as a process on $(Y,{{\,\mathrm{\mathbb {P}}\,}})$. Then

$$\begin{aligned} W_n\rightarrow _w L_\alpha \;\text {in }D([0,1],{\mathbb {R}}^d)\text { with the } {\mathcal {SM}}_1\text { topology as }n\rightarrow \infty , \end{aligned}$$

where $L_\alpha$ is a *d*-dimensional $\alpha$-stable Lévy process.

### Remark 1.2

The limiting process $L_\alpha$ is explicitly identified in Sect. 6.2.

In the context of [16], the conclusion $W_n\rightarrow _w L_\alpha$ was sufficient to prove the homogenisation result $X_n\rightarrow _w X$. This is not the case for general noise, and we require tightness in *p*-variation. For $1 \le p<\infty$, recall that the *p-variation* of $u:[0,1]\rightarrow {\mathbb {R}}^d$ is given by

1.6 $$\begin{aligned} \Vert u\Vert _{p\text {-}\mathrm {var}} = \sup _{0=t_0<t_1<\dots <t_k=1} \left( \sum _{j=1}^k\bigl |u(t_j) - u(t_{j-1})\bigr |^p\right) ^{1/p} , \end{aligned}$$

where $| \cdot |$ denotes the Euclidean norm on ${\mathbb {R}}^d$.

### Theorem 1.3

Consider the intermittent map (1.4) or (1.5) with $\alpha \in (1,2)$ and let $v:Y\rightarrow {\mathbb {R}}^d$ be Hölder with $\int _Y v\mathrm {d}\mu =0$. Let ${{\,\mathrm{\mathbb {P}}\,}}$ be any probability measure on *Y* that is absolutely continuous with respect to Lebesgue. Then the family of random variables $\Vert W_n\Vert _{p\text {-}\mathrm {var}}$ is tight on $(Y,{{\,\mathrm{\mathbb {P}}\,}})$ for all $p>\alpha$.

The main abstract result in this paper states that the properties established in Theorems 1.1 and 1.3 are the key ingredients required to solve the homogenisation problem. Informally:

Consider the fast–slow system (1.1) and define $W_n$ as in (1.2) and $X_n=x_{\lfloor nt \rfloor }^{(n)}$ with $x^{(n)}_0=\xi _n$. Suppose that $\lim _{n\rightarrow \infty }\xi _n = \xi$, $W_n\rightarrow _w L_\alpha$, an $\alpha$-stable Lévy process, in $D([0,1],{\mathbb {R}}^d)$ with the ${\mathcal {SM}}_1$-topology, and that $\Vert W_n\Vert _{p\text {-}\mathrm {var}}$ is tight for all $p>\alpha$.

If *v* is bounded and $a,\,b$ are sufficiently smooth, then $X_n\rightarrow _w X$ in $D([0,1],{\mathbb {R}}^m)$ where *X* is the solution to the SDE (1.3).

We give a rigorous formulation of this result in Theorem 2.6 (in the above statement we assume that the limiting process is Lévy only for convenience—the result holds true for an arbitrary limiting process as seen from Theorem 2.6). To complete the statement, it is necessary to describe the topology on $D([0,1],{\mathbb {R}}^m)$ in which $X_n$ converges. As already indicated, the ${\mathcal {SM}}_1$ topology is too strong in general. The next example illustrates where the problem lies.

### Example 1.4

Let $\theta > 0$ and consider continuous deterministic processes $W_n :[0,1] \rightarrow {\mathbb {R}}$ which are equal to 0 on $[0,\frac{1}{2}]$, equal to $\theta$ on $[\frac{1}{2} + \frac{1}{n}, 1]$, and linear on $[\frac{1}{2}, \frac{1}{2} + \frac{1}{n}]$. Let $X_n = (X_n^1, X_n^2)$ be the solution of the ordinary differential equation

$$\begin{aligned} \begin{pmatrix} \mathrm {d}X_n^1 \\ \mathrm {d}X_n^2 \end{pmatrix} = \begin{pmatrix} - X_n^2 \\ X_n^1 \end{pmatrix} \mathrm {d}W_n , \qquad \begin{pmatrix} X_n^1(0) \\ X_n^2(0) \end{pmatrix} = \begin{pmatrix} 1 \\ 0 \end{pmatrix} . \end{aligned}$$

**Fig. 2:**
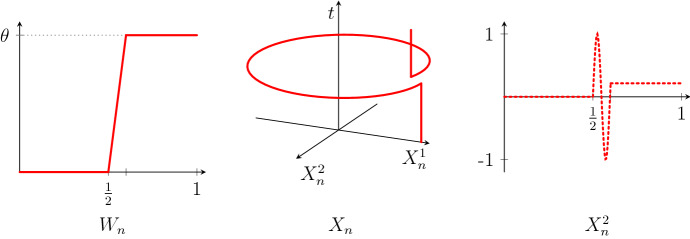
Graphs of $W_n$ and $X_n=(X_n^1,X_n^2)$ in Example 1.4

The graphs of $W_n$ and $X_n$ are shown in Fig. 2.

It is easy to see that $W_n$ converges to $\theta \, 1_{[1/2,1]}$ in the ${\mathcal {M}}_1$ topology as $n \rightarrow \infty$, and that $(X_n^1, X_n^2) = (\cos W_n , \sin W_n)$. The process $X_n$ converges pointwise to

$$\begin{aligned} X(t) = {\left\{ \begin{array}{ll} (1,0), &{}\quad t \le \frac{1}{2}, \\ (\cos \theta , \sin \theta ), &{}\quad t > \frac{1}{2} . \end{array}\right. } \end{aligned}$$

In particular, if $\theta = 2 \pi$, then $X \equiv (1,0)$ is continuous. At the same time, $X_n$ fails to converge in any of the Skorokhod topologies.

**Fig. 3:**
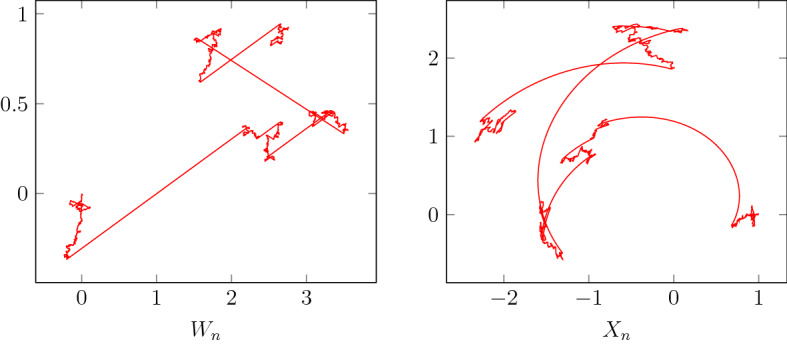
Realisation of $W_n$ and $X_n$ with $n=10^4$ points

The problem outlined in Example 1.4 arises naturally in the fast–slow system (1.1). Figure 3 illustrates a realisation^1^ of $W_n$ and $X_n$ for $d=m=2$ and the map (1.5). The function *b* is taken as

$$\begin{aligned} b(x_1,x_2)\left( {\begin{array}{c}v_1\\ v_2\end{array}}\right) = \left( {\begin{array}{c}-x_2\\ x_1\end{array}}\right) v_1 + \left( {\begin{array}{c}x_1\\ x_2\end{array}}\right) v_2 . \end{aligned}$$

Note that, although $W_n$ appears to converge in ${\mathcal {SM}}_1$ in accordance with Theorem 1.1, $X_n$ moves along the integral curves of a vector field, and thus does not approximate its limit in ${\mathcal {SM}}_1$.

Topologies naturally suited for convergence in Example 1.4 were recently introduced in [7]. These topologies are a generalisation of the Skorokhod ${\mathcal {SM}}_1$ topology which allow for convenient control of differential equations. Briefly, jumps of a càdlàg process are interpreted as an instant travel along prescribed continuous paths which depend only on the start and end points of the jump. The full “pathspace” thus becomes the set of pairs $(X,\phi )$, where $X:[0,1]\rightarrow {\mathbb {R}}^d$ is a càdlàg path and $\phi$ is a so-called *path function* [6] which maps each jump $(X(t-),X(t))$ to a continuous path from $X(t-)$ to *X*(*t*). It is often convenient to fix $\phi$, which in turn determines a topology on càdlàg paths; if $\phi$ is linear, one recovers the ${\mathcal {SM}}_1$ topology. For our purposes, it is necessary to adapt the spaces introduced in [7], and we give details in Sects. 2 and 3.

The paper is organised as follows. In Sect. 2, we introduce the necessary prerequisites on generalised Skorokhod topologies and Marcus differential equations in order to state rigorously our main abstract result Theorem 2.6. The proof is given at the end of Sect. 3 after introducing the necessary results from rough path theory. In Sects. 4 to 6, we show that a class of nonuniformly expanding dynamical systems, including (1.4) and (1.5), satisfies the conclusions of Theorems 1.1 and 1.3 which are in turn the main hypotheses of Theorem 2.6. Section 4 deals with a class of uniformly expanding maps known as Gibbs–Markov maps, and Sect. 5 provides the inducing step to pass from uniformly expanding maps to nonuniformly expanding maps. In Sect. 6, we apply the results of Sects. 4 and 5 to the intermittent maps (1.4) and (1.5). The precise result on homogenisation of the system (1.1) with fast dynamics given by either (1.4) or (1.5) is stated in Corollary 6.4.

Notation We use “big O” and $\lesssim$ notation interchangeably, writing $a_n=O(b_n)$ or $a_n\lesssim b_n$ if there is a constant $C>0$ such that $a_n\le Cb_n$ for all sufficiently large *n*. As usual, $a_n=o(b_n)$ means that $\lim _{n\rightarrow \infty }a_n/b_n=0$ and $a_n\sim b_n$ means that $\lim _{n\rightarrow \infty }a_n/b_n=1$.

## Setup and result

In this section, we collect the material necessary to formulate our main abstract result Theorem 2.6.

### Skorokhod topologies

Let $D = D([0,1],{\mathbb {R}}^d)$ denote the Skorokhod space of càdlàg functions, i.e. the set of functions $X :[0,1] \rightarrow {\mathbb {R}}^d$ which are right-continuous with left limits. For $X\in D$ and $t\in [0,1]$, we denote $X(t-) = \lim _{s \nearrow t} X(s)$, with the convention that $X(0-) = X(0)$.

Let $\Lambda$ denote the set of all increasing bijections $\lambda :[0,1] \rightarrow [0,1]$ and let ${\mathrm {id}}\in \Lambda$ denote the identity map ${\mathrm {id}}(t)=t$. For $X_1, X_2 \in D$, let ${\varvec{\sigma }}_\infty (X_1, X_2)$ be the Skorokhod distance

$$\begin{aligned} {\varvec{\sigma }}_\infty (X_1, X_2) = \inf _{\lambda \in \Lambda } \max \{ \Vert \lambda - {\mathrm {id}}\Vert _\infty , \Vert X_1 \circ \lambda - X_2\Vert _\infty \}, \end{aligned}$$

where $\Vert X\Vert _\infty = \sup _{t\in [0,1]}|X(t)|$. The topology on *D* induced by ${\varvec{\sigma }}_\infty$ is known as the strong ${\mathcal {J}}_1$, or ${\mathcal {SJ}}_1$, topology.

Another important topology on *D* is the strong ${\mathcal {M}}_1$, or ${\mathcal {SM}}_1$, topology defined as follows. For $X\in D$ consider the “completed” graph $\Gamma (X) = \{(t,x) \in [0,1]\times {\mathbb {R}}^d : x\in [X(t-),X(t)]\}$, and let $\Lambda ^*(X)$ be the set of all continuous bijections $(\lambda ,\gamma ) :[0,1] \rightarrow \Gamma (X)$ with $\lambda (0)=0$. Then the ${\mathcal {SM}}_1$ topology on *D* is induced by the metric

$$\begin{aligned} d_{{\mathcal {SM}}_1}(X_1, X_2) = \inf _{\begin{array}{c} (\lambda _i,\gamma _i)\in \Lambda ^*(X_i)\\ i=1,2 \end{array}} \max \{\Vert \lambda _1-\lambda _2\Vert _\infty , \Vert \gamma _1- \gamma _2\Vert _\infty \} . \end{aligned}$$

### Generalised ${\mathcal {SM}}_1$ topologies

We now introduce generalisations of the ${\mathcal {SM}}_1$ topology from [7].

For $1 \le p<\infty$, recall the *p*-variation $\Vert u\Vert _{p\text {-}\mathrm {var}}$ of $u:[0,1]\rightarrow {\mathbb {R}}^d$ defined by (1.6). We furthermore denote . Let

$$\begin{aligned} D^{p\text {-}\mathrm {var}} = \big \{u\in D\big ([0,1],{\mathbb {R}}^{d}\big ) : \Vert u\Vert _{p\text {-}\mathrm {var}}<\infty \big \} \end{aligned}$$

and $C^{p\text {-}\mathrm {var}}([0,1],{\mathbb {R}}^d)\subset D^{p\text {-}\mathrm {var}}$ be the set of $u\in D^{p\text {-}\mathrm {var}}$ which are continuous. Let ${\varvec{\sigma }}_{p\text {-}\mathrm {var}}$ denote the Skorokhod-type *p*-variation on $D^{p\text {-}\mathrm {var}}$:

#### Definition 2.1

A *path function* on ${\mathbb {R}}^d$ is a map $\phi :J \rightarrow C([0,1], {\mathbb {R}}^d)$, where $J \subset {\mathbb {R}}^d\times {\mathbb {R}}^d$, for which $\phi (x,y)(0) = x$ and $\phi (x,y)(1) = y$ for all $(x,y)\in J$. For a path $X \in D([0,1], {\mathbb {R}}^d)$, we say that $t\in [0,1]$ is a jump time of *X* if $X(t-) \ne X(t)$. A pair $(X,\phi )$ is called admissible if all the jumps of *X* are in the domain of definition of $\phi$, i.e. $(X(t-),X(t)) \in J$ for all jump times *t* of *X*. We denote by ${\bar{\mathscr {D}}}([0,1],{\mathbb {R}}^d)$ the space of admissible pairs $(X, \phi )$. We let ${\mathscr {D}}([0,1],{\mathbb {R}}^d) = {\bar{\mathscr {D}}}([0,1],{\mathbb {R}}^d) / \sim$, where $(X_1, \phi _1) \sim (X_2, \phi _2)$ if $X_1 = X_2$ and $\phi _1(X_1(t-),X_1(t))$ is a reparametrisation of $\phi _2(X_1(t-),X_1(t))$ for all jump times *t* of $X_1$.

#### Remark 2.2

We often keep implicit the interval [0, 1] and ${\mathbb {R}}^d$, as well as *J*, when they are clear from the context. We allow *J* to be a strict subset of ${\mathbb {R}}^d\times {\mathbb {R}}^d$ since this case arises naturally when considering driver–solution pairs for canonical differential equations, see the final discussion in Sect. 2.3.

A simple path function which shall play an important role is the following.

#### Definition 2.3

The *linear path function* on ${\mathbb {R}}^{k}$ is the map $\ell _k:{\mathbb {R}}^k\times {\mathbb {R}}^k \rightarrow C([0,1],{\mathbb {R}}^k)$ defined by $\ell _k(x,y)(t) = x+t(y-x)$ for all $x,y\in {\mathbb {R}}^{k}$.

Fix a sequence $r_1, r_2, \ldots > 0$ with $\sum _j r_j < \infty$. Given $(X, \phi ) \in {\bar{\mathscr {D}}}$ and $\delta > 0$, let $X^{\phi , \delta } \in C([0,1], {\mathbb {R}}^d)$ denote the continuous version of *X*, where the *k*-th largest jump is made continuous using $\phi$ on a fictitious time interval of length $\delta r_k$. More precisely:

- Let $m\ge 0$ be the number of jumps (possibly infinite) of *X*. We order the jump times $\{t_j\}_{j=1}^m$ so that $|X(t_k) - X(t_k-)| \ge |X(t_{k+1}) - X(t_{k+1}-)|$ for each *k*, with $t_k < t_{k+1}$ in case of equality.
- Let $r= \sum _{j=1}^m r_j$ and define the map
  2.1 $$\begin{aligned} \tau :[0,1] \rightarrow [0, 1+\delta r], \quad \tau (t) = t + \sum _k \delta r_k 1_{\{t_k \le t\}} . \end{aligned}$$
- Define an intermediate process ${\hat{X}}\in C([0,1+\delta r], {\mathbb {R}}^d)$,
  $$\begin{aligned} {\hat{X}}(t) = {\left\{ \begin{array}{ll} X(s) &{}\quad \text {if } t = \tau (s) \text { for some } s \in [0,1], \\ \phi (X(t_k-), X(t_k))\bigl (\frac{s - \tau (t_k-)}{\delta r_k}\bigr ) &{} \quad \text {if } t \in [\tau (t_k-), \tau (t_k)) \text { for some } k . \end{array}\right. } \end{aligned}$$
- Finally, let $X^{\phi , \delta }(t) = {\hat{X}}(t (1+\delta r))$, scaling the domain of ${\hat{X}}$ from $[0, 1+\delta r]$ to [0, 1].

For $(X, \phi ) \in {\mathscr {D}}([0,1],{\mathbb {R}}^{d})$ and $p \ge 1$, let

$$\begin{aligned} \Vert (X, \phi )\Vert _{p\text {-}\mathrm {var}} = \Vert X^{\phi , 1}\Vert _{p\text {-}\mathrm {var}} . \end{aligned}$$

Note that $\Vert (X, \phi )\Vert _{p\text {-}\mathrm {var}}$ is well-defined since $\Vert X^{\phi , 1}\Vert _{p\text {-}\mathrm {var}}$ depends on neither the parametrisation of $\phi$, nor the sequence $\{r_k\}$. Let

$$\begin{aligned} {\mathscr {D}}^{p\text {-}\mathrm {var}} = \{ (X, \phi ) \in {\mathscr {D}}: \Vert (X, \phi )\Vert _{p\text {-}\mathrm {var}} < \infty \} . \end{aligned}$$

Given $(X_1, \phi _1)$ and $(X_2, \phi _2)$ in ${\mathscr {D}}^{p\text {-}\mathrm {var}}$, let

$$\begin{aligned} {\varvec{\alpha }}_{p\text {-}\mathrm {var}} ((X_1, \phi _1), (X_2, \phi _2)) = \lim _{\delta \rightarrow 0} {\varvec{\sigma }}_{p\text {-}\mathrm {var}} \big (X_1^{\phi _1, \delta }, X_2^{\phi _2, \delta }\big ), \end{aligned}$$

which defines a metric on ${\mathscr {D}}^{p\text {-}\mathrm {var}}$ [7, Remark 3.8].

### Marcus differential equations

For $\gamma >0$, let $C^\gamma ({\mathbb {R}}^m,{\mathbb {R}}^n)$ denote the space of functions $b :{\mathbb {R}}^m \rightarrow {\mathbb {R}}^n$ such that

$$\begin{aligned} \Vert b\Vert _{C^\gamma } = \max _{|\alpha |=0,\ldots ,\lfloor \gamma \rfloor } \Vert D^\alpha b\Vert _\infty + \sup _{x,y\in {\mathbb {R}}^m} \max _{|\alpha |=\lfloor \gamma \rfloor } \frac{|D^\alpha b(x) - D^\alpha b(y)|}{|x-y|^{\gamma -\lfloor \gamma \rfloor }} < \infty . \end{aligned}$$

Note that our notation is slightly non-standard since $b\in C^N$ for $N\in {\mathbb {N}}$ implies only that the $(N-1)$-th derivative of *b* is Lipschitz rather than continuous.

Suppose that $W \in D^{p\text {-}\mathrm {var}}([0,1], {\mathbb {R}}^{d})$ with $1\le p < 2$, and that $a\in C^\beta ({\mathbb {R}}^m,{\mathbb {R}}^m)$ and $b \in C^{\gamma }({\mathbb {R}}^m,{\mathbb {R}}^{m\times d})$ with $\beta > 1$ and $\gamma > p$. Under these conditions, we can define and solve (in a purely deterministic way) a Marcus-type differential equation

2.2 $$\begin{aligned} \mathrm {d}X = a(X) \mathrm {d}t + b(X) \diamond \mathrm {d}W . \end{aligned}$$

The solution is obtained as follows from the theory of continuous rough differential equations (RDEs) in the Young regime [12, 14, 28]. Consider the càdlàg path ${\widetilde{W}}:[0,1]\rightarrow {\mathbb {R}}^{1+d}$ given by ${\widetilde{W}}(t) = (t,W(t))$. Using the notation of Sect. 2.2, consider the continuous path ${\widetilde{W}}^{\phi ,1} :[0,1+r] \rightarrow {\mathbb {R}}^d$, where $\phi =\ell _{1+d}$ is the linear path function on ${\mathbb {R}}^{1+d}$. Let $\tau :[0,1] \rightarrow [0,1+r]$ be the corresponding map given by (2.1). Then $\Vert {\widetilde{W}}^{\phi ,1}\Vert _{p\text {-}\mathrm {var}}\lesssim \Vert W\Vert _{p\text {-}\mathrm {var}}$ (see e.g. [6, Corollary A.6]), and therefore one can solve the (continuous) RDE

$$\begin{aligned} \mathrm {d}{\widetilde{X}}= (a,b)({\widetilde{X}}) \mathrm {d}{\widetilde{W}}. \end{aligned}$$

The solution is a continuous path ${\widetilde{X}}:[0,1+r] \rightarrow {\mathbb {R}}^m$ of finite *p*-variation. The solution to (2.2) is the càdlàg path $X :[0,1]\rightarrow {\mathbb {R}}^d$ given by $X(t) = {\widetilde{X}}(\tau (t))$. We discuss a more general interpretation of this equation in Sect. 3.2.

#### Remark 2.4

In the case that *W* is a semimartingale, one can verify that *X* is the solution to the classical Marcus SDE (see [7, Proposition 4.16] for the general case $p>2$ but with stronger regularity assumptions on *a*, *b*; the proof carries over to our setting without change).

To properly describe solutions of (2.2) and regularity of the solution map $W \mapsto X$, it is not enough to look at *X* as an element of $D([0,1],{\mathbb {R}}^m)$. As in Example 1.4, one may have $X \equiv 0$ say, but with sizeable jumps in fictitious time.

Following [7], we consider the driver-solution space $D([0,1], {\mathbb {R}}^{d + m})$, made to contain the pairs (*W*, *X*), and introduce a new path function on ${\mathbb {R}}^{d+m}$.

#### Definition 2.5

Consider $b\in C^{1}({\mathbb {R}}^m,{\mathbb {R}}^{m\times d})$. For $x \in {\mathbb {R}}^m$ and $\Phi \in C^{1\text {-}\mathrm {var}}([0,1],{\mathbb {R}}^{d})$, let $\pi _b[x;\Phi ] \in C^{1\text {-}\mathrm {var}}([0,1], {\mathbb {R}}^m)$ denote the solution $\Pi$ of the equation

$$\begin{aligned} \mathrm {d}\Pi = b(\Pi ) \mathrm {d}\Phi , \quad \Pi (0) = x . \end{aligned}$$

We define the path function $\phi _b$ on ${\mathbb {R}}^{d+m}$ by

2.3 $$\begin{aligned} \phi _b\bigl ((w_1,x_1), (w_2,x_2) \bigr )(t) = \bigl (\ell _d(w_1,w_2)(t), \pi _b[x_1;\ell _{d}(w_1,w_2)](t)\bigr ) , \end{aligned}$$

which is defined on

$$\begin{aligned} J_b = \bigl \{\bigl ((w_1,x_1), (w_2,x_2) \bigr ) : w_1,w_2\in {\mathbb {R}}^{d},\; \pi _b[x_1;\ell _{d}(w_1,w_2)](1) = x_2 \bigr \} . \end{aligned}$$

Note that $J_b$ is a strict subset of ${\mathbb {R}}^{d+m}\times {\mathbb {R}}^{d+m}$. Observe that if *X* solves (2.2), then $((W,X),\phi _b)\in {\mathscr {D}}^{p\text {-}\mathrm {var}}([0,1],{\mathbb {R}}^{d+m})$ and the path function $\phi _b$ describes how the discontinuities of (*W*, *X*) are traversed in fictitious time.

### Main abstract result

Now we are ready for a rigorous formulation of the main abstract result. Consider the fast–slow system (1.1) with initial condition $x^{(n)}_0 = \xi _n$ such that $\lim _{n\rightarrow \infty }\xi _n = \xi$. Suppose that $\alpha \in (1,2)$, $\alpha '\in [\alpha ,2)$, $v\in L^\infty (Y,{\mathbb {R}}^d)$, $a\in C^{\beta }({\mathbb {R}}^m,{\mathbb {R}}^m)$, $b \in C^\gamma ({\mathbb {R}}^m,{\mathbb {R}}^{m\times d})$ for some $\beta >1$, $\gamma > \alpha '$. Define $W_n$ as in (1.2) and $X_n(t) = x_{\lfloor nt \rfloor }^{(n)}$.

#### Theorem 2.6

Suppose that

- $W_n\rightarrow _w L$ in $D([0,1],{\mathbb {R}}^d)$ with the ${\mathcal {SM}}_1$ topology as $n\rightarrow \infty$ for some process *L*.
- $\Vert W_n\Vert _{p\text {-}\mathrm {var}}$ is tight for all $p>\alpha '$.

Then, for all $p>\alpha '$, it holds that $\Vert L\Vert _{p\text {-}\mathrm {var}}<\infty$ a.s. and

$$\begin{aligned} ((W_n, X_n), \ell _{d+m}) \rightarrow _w ((L, X), \phi _{b}) \qquad \text {as} \qquad n \rightarrow \infty \end{aligned}$$

in $({\mathscr {D}}^{p\text {-}\mathrm {var}}([0,1],{\mathbb {R}}^{d +m}), {\varvec{\alpha }}_{p\text {-}\mathrm {var}})$, where *X* is the solution of the Marcus differential equation

2.4 $$\begin{aligned} \mathrm {d}X = a(X) \mathrm {d}t + b(X) \diamond \mathrm {d}L , \qquad X(0) = \xi \in {\mathbb {R}}^m . \end{aligned}$$

The proof of Theorem 2.6 is given at the end of Sect. 3.

#### Remark 2.7

- The property $\Vert L\Vert _{p\text {-}\mathrm {var}}<\infty$ a.s. together with $\gamma >\alpha '$ guarantees that the Marcus equation (2.4) admits a unique solution for a.e. realisation of *L*. In our applications, *L* is an $\alpha$-stable Lévy process, for which the finiteness of $\Vert L\Vert _{p\text {-}\mathrm {var}}$ is classical, and we take $\alpha '=\alpha$. We introduce the parameter $\alpha '$ to highlight that the threshold for the value of *p* in the second condition of Theorem 2.6 does not need to be the same $\alpha$ as in (1.2).
- The drift vector field *a* plays no role in the definition of $\phi _b$. This is expected since the driver $V_n(t)=n^{-1}\lfloor tn \rfloor$ corresponding to *a* in the RDE solved by $X_n$ (see the proof of Theorem 2.6 below) converges in *q*-variation for every $q>1$ to a process with no jumps.
- Since the limiting process *L* in general has jumps, it is crucial that we pair (*L*, *X*) with the path function $\phi _b$. In contrast, the jumps of $(W_n,X_n)$ are of magnitude at most $n^{-1/\alpha }$, so $(W_n,X_n)$ is almost a continuous path for large *n*;
  we make the reference to $\ell _{d+m}$ only for convenience (cf. (3.10) below).

Recall that a stochastic process $(L_t)_{t \in [0,1]}$ is called *stochastically continuous* if, for all $t\in [0,1]$, $L_s\rightarrow L_t$ in probability as $s\rightarrow t$. Note that Lévy processes are stochastically continuous by definition.

#### Corollary 2.8

In the setting of Theorem 2.6, suppose further that the process *L* is stochastically continuous. Then $X_n \rightarrow X$ in the sense of finite dimensional distributions.

#### Proof

Consider $0 \le t_1< \cdots < t_k \le 1$. The map

2.5 $$\begin{aligned} (Y, \phi ) \mapsto (Y(t_1), \ldots , Y(t_k)) , \qquad \big ({\mathscr {D}}^{p\text {-}\mathrm {var}}([0,1],{\mathbb {R}}^{d+m}), {\varvec{\alpha }}_{p\text {-}\mathrm {var}}\big ) \rightarrow {\mathbb {R}}^{(d+m)k} \end{aligned}$$

is continuous at $(Y,\phi )$ whenever the path *Y* is continuous at all $t_j$, see [7, Lemma 2.12]. Furthermore, if $t\in [0,1]$ is a continuity point of *L*, then it is also a continuity point of the solution *X* to (2.4). Since *L* is càdlàg and stochastically continuous, any fixed $t\in [0,1]$ is a.s. a continuity point of *L* (see e.g. the proof of [2, Lemma 2.3.2]), $((L,X),\phi _b)$ is a.s. a continuity point of the map (2.5). In particular, by Theorem 2.6 and the continuous mapping theorem, $(X_n(t_1), \ldots , X_n(t_k))$ converges in law to $(X(t_1), \ldots , X(t_k))$, as required. $\square$

#### Remark 2.9

As in Example 1.4, we do not expect that $X_n \rightarrow _w X$ in any of the Skorokhod topologies, or that $f(X_n) \rightarrow _w f(X)$ for certain standard functionals $f :D \rightarrow {\mathbb {R}}$ that are continuous with respect to the Skorokhod topologies, such as $f(X) = \Vert X\Vert _\infty$. Instead we have for example that $\Vert {\widetilde{X}}_n\Vert _\infty \rightarrow _w \Vert {\widetilde{X}}\Vert _\infty$, where ${\widetilde{X}}_n$ and ${\widetilde{X}}$ are the corresponding components of the continuous paths $(W_n, X_n)^{\ell _{d+m}, 1}$ and $(W, X)^{\phi _b, 1}$.

## Rough path formulation

In this section we expand the material in Sect. 2 in order to formulate and prove an abstract convergence result, Theorem 3.4, from which Theorem 2.6 follows.

### Generalised ${\mathcal {SM}}_1$ topologies with mixed variation

We use a modified version of the topologies from [7] suitable for handling differential equations with drift. We continue using notation from Sect. 2.

For $1\le p,q<\infty$, we define the mixed (*q*, *p*)-variation for $u = (u^0,u^1,\ldots , u^d) = (u^0,\bar{u}) :[0,1]\rightarrow {\mathbb {R}}^{1+d}$ by

$$\begin{aligned} \Vert u\Vert _{(q,p)\text {-}\mathrm {var}} = \Vert u^0\Vert _{q\text {-}\mathrm {var}} + \Vert \bar{u}\Vert _{p\text {-}\mathrm {var}} . \end{aligned}$$

Let

$$\begin{aligned} D^{(q,p)\text {-}\mathrm {var}} = \big \{u\in D\big ([0,1],{\mathbb {R}}^{1+d}\big ) : \Vert u\Vert _{(q,p)\text {-}\mathrm {var}}<\infty \big \} \end{aligned}$$

and $C^{(q,p)\text {-}\mathrm {var}}([0,1],{\mathbb {R}}^{1+d})\subset D^{(q,p)\text {-}\mathrm {var}}$ be the set of $u\in D^{(q,p)\text {-}\mathrm {var}}$ which are continuous. We furthermore denote  and defineGiven $(X_1, \phi _1)$ and $(X_2, \phi _2)$ in ${\bar{\mathscr {D}}}$, let

$$\begin{aligned} {\varvec{\alpha }}_\infty ((X_1, \phi _1), (X_2, \phi _2)) = \lim _{\delta \rightarrow 0} {\varvec{\sigma }}_\infty \big (X_1^{\phi _1, \delta }, X_2^{\phi _2, \delta }\big ) . \end{aligned}$$

Following [7, Lemma 2.7], the limit exists, is independent of the choice of the sequence $r_k$, and is invariant under reparametrisation of the path functions. In particular, ${\varvec{\alpha }}_\infty$ induces a pseudometric on ${\mathscr {D}}$.

For $$(X, \phi ) \in {\mathscr {D}}([0,1],{\mathbb {R}}^{1+d})$$, let

$$\begin{aligned} \Vert (X, \phi )\Vert _{(q,p)\text {-}\mathrm {var}} = \Vert X^{\phi , 1}\Vert _{(q,p)\text {-}\mathrm {var}} . \end{aligned}$$

As before, note that $\Vert (X, \phi )\Vert _{(q,p)\text {-}\mathrm {var}}$ is well-defined since $\Vert X^{\phi , 1}\Vert _{(q,p)\text {-}\mathrm {var}}$ does not depend on the parametrisation of $\phi$, nor the sequence $\{r_k\}$. Let

$$\begin{aligned} {\mathscr {D}}^{(q,p)\text {-}\mathrm {var}} = \{ (X, \phi ) \in {\mathscr {D}}: \Vert (X, \phi )\Vert _{(q,p)\text {-}\mathrm {var}} < \infty \} . \end{aligned}$$

Given $(X_1, \phi _1)$ and $(X_2, \phi _2)$ in ${\mathscr {D}}^{(q,p)\text {-}\mathrm {var}}$, let

$$\begin{aligned} {\varvec{\alpha }}_{(q,p)\text {-}\mathrm {var}} ((X_1, \phi _1), (X_2, \phi _2)) = \lim _{\delta \rightarrow 0} {\varvec{\sigma }}_{(q,p)\text {-}\mathrm {var}} \big (X_1^{\phi _1, \delta }, X_2^{\phi _2, \delta }\big ), \end{aligned}$$

which is well-defined and induces a metric on ${\mathscr {D}}^{(q,p)\text {-}\mathrm {var}}$ (cf. [7, Remark 3.8]).

### Differential equations with càdlàg drivers

For $\beta ,\gamma >0$, denote by $C^{\beta ,\gamma }$ the space of all $b=(b^0,b^1,\ldots , b^d) :{\mathbb {R}}^m \rightarrow {\mathbb {R}}^{m \times (1+d)}$ such that

$$\begin{aligned} \Vert b\Vert _{C^{\beta ,\gamma }} = \Vert b^0\Vert _{C^{\beta }} + \max _{i=1,\ldots , d}\Vert b^i\Vert _{C^{\gamma }} < \infty . \end{aligned}$$

Suppose $1\le q \le p < 2$ and that $b \in C^{\beta ,\gamma }$ with $\beta >q$ and $\gamma > p$ such that

3.1 $$\begin{aligned} \frac{\beta -1}{p} + \frac{1}{q}> 1 \quad \text { and } \quad \frac{\gamma -1}{q} + \frac{1}{p} > 1 . \end{aligned}$$

#### Remark 3.1

See [14, Remark 12.7] for a discussion about condition (3.1). In our applications, we will consider $\beta >1$ and $\gamma >p$ as fixed, and $q=1+\kappa$ for $\kappa >0$ arbitrarily small. In this case condition (3.1) is always attained by taking $\kappa$ sufficiently small, which explains why it does not appear in Theorem 2.6.

Recall that under these conditions, if $W \in C^{(q,p)\text {-}\mathrm {var}}([0,1],{\mathbb {R}}^{1+d})$, then the canonical RDE (in the Young regime)

$$\begin{aligned} \mathrm {d}X = b(X) \mathrm {d}W ,\qquad X(0) = \xi \in {\mathbb {R}}^m \end{aligned}$$

admits a unique solution $X\in C^{p\text {-}\mathrm {var}}([0,1],{\mathbb {R}}^m)$.

For general $W\in D^{(q,p)\text {-}\mathrm {var}}([0,1],{\mathbb {R}}^{1+d})$, consider the RDE

3.2 $$\begin{aligned} \mathrm {d}X = b(X) * \mathrm {d}W ,\qquad X(0) = \xi . \end{aligned}$$

Here, $*$ stands for one of the different ways to interpret a differential equation in the presence of discontinuities, which in general result in different solutions *X*. Two common choices (considered in the case $q=p$ by Williams [44] and studied further in [6, 7, 13, 15]) are

- *Geometric (Marcus) RDE.* The solution is completely analogous to that of (2.2): we solve the continuous RDE $\mathrm {d}{\widetilde{X}}= b({\widetilde{X}})\mathrm {d}W^{\phi ,1}$, where $\phi =\ell _{1+d}$ is the linear path function on ${\mathbb {R}}^{1+d}$, and then remove the fictitious time intervals (note that the RDE is well-posed since $\Vert W^{\phi ,1}\Vert _{(q,p)\text {-}\mathrm {var}} \lesssim \Vert W\Vert _{(q,p)\text {-}\mathrm {var}}$ by Chevyrev [6, Corollary A.6]). For geometric RDEs we use the notation
  3.3 $$\begin{aligned} \mathrm {d}X = b(X) \diamond \mathrm {d}W ,\qquad X(0) = \xi . \end{aligned}$$
  Observe that $((W,X),\phi _b)\in {\mathscr {D}}^{(q,p)\text {-}\mathrm {var}}([0,1],{\mathbb {R}}^{1+d+m})$, where $\phi _b$ is the path function on ${\mathbb {R}}^{1+d+m}$ as in Definition 2.5 with $\ell _{d}$ replaced by $\ell _{1+d}$.
- *Forward (Itô) RDE.* The solution satisfies the integral equation
  3.4 $$\begin{aligned} X(t) = X(0) + \int _0^t b(X(s-)) \mathrm {d}W(s), \end{aligned}$$
  where the integral is understood as a limit of Riemann–Stieltjes sums with $b(X(s-))$ evaluated at the left limit points of the partition intervals:
  $$\begin{aligned} \int _0^t b(X(s-)) \mathrm {d}W(s) = \lim _{|{\mathcal {P}}| \rightarrow 0} \sum _{[s,s'] \in {\mathcal {P}}} b(X(s-)) (W(s') - W(s)) . \end{aligned}$$
  Here, ${\mathcal {P}}$ are partitions of [0, *t*] into intervals, and $|{\mathcal {P}}|$ is the size of the longest interval. For forward RDEs we use the notation
  $$\begin{aligned} \mathrm {d}X = b(X)^- \mathrm {d}W ,\qquad X(0) = \xi . \end{aligned}$$

#### Remark 3.2

Geometric RDEs use linear paths to connect the endpoints of each jump. As mentioned in the introduction, this has been generalised in [7] allowing one to solve

3.5 $$\begin{aligned} \mathrm {d}X = b(X) \diamond \mathrm {d}(W, \phi ) ,\qquad X(0) = \xi , \end{aligned}$$

for any $(W,\phi ) \in {\mathscr {D}}^{(q,p)\text {-}\mathrm {var}}([0,1],{\mathbb {R}}^{1+d})$. The interpretation is as for geometric RDEs: we construct a continuous path, solve the canonical RDE $\mathrm {d}{\widetilde{X}}= b({\widetilde{X}}) \mathrm {d}W^{\phi ,1}$, and then remove fictitious time intervals. Then $((W,X), \phi _b) \in {\mathscr {D}}^{(q,p)\text {-}\mathrm {var}}([0,1], {\mathbb {R}}^{1+d+m})$, where $\phi _b$ is the path function on ${\mathbb {R}}^{1+d+m}$ as in Definition 2.5 with $\ell _{d}$ replaced by $\phi$, and the solution map of (3.5)

$$\begin{aligned} {\mathbb {R}}^m \times \bigl ({\mathscr {D}}^{(q,p)\text {-}\mathrm {var}}([0,1], {\mathbb {R}}^{1+d}), {\varvec{\alpha }}_{(q,p)\text {-}\mathrm {var}}\bigr )&\rightarrow \bigl ({\mathscr {D}}^{(q,p)\text {-}\mathrm {var}}([0,1], {\mathbb {R}}^{1+d+m}), {\varvec{\alpha }}_{(q,p)\text {-}\mathrm {var}}\bigr ) , \\ (\xi , (W, \phi ))&\mapsto ((W, X), \phi _b) \end{aligned}$$

is locally Lipschitz continuous. (These results were shown in [7, Theorem 3.13] for $q=p$, but the same proof applies mutatis mutandis for the general case upon using the RDE with drift estimates [14, Theorem 12.10]. In fact one can allow rough path drivers in ${\mathbb {R}}^{d'+d}$ with finite (*q*, *p*)-variation for arbitrary $p,q\ge 1$ satisfying $p^{-1}+q^{-1}>1$. We consider only $d'=1$ and $1\le q\le p<2$ since this suffices for our purposes.)

### Convergence of forward RDEs to geometric RDEs

For the remainder of this section, let us fix $1\le q\le p<2$, $\beta >q$, $\gamma >p$, such that (3.1) holds. Suppose that $W \in D^{(q,p)\text {-}\mathrm {var}}([0,1],{\mathbb {R}}^{1+d})$ and $b \in C^{\beta ,\gamma }$. Then for every $\xi \in {\mathbb {R}}^m$, the geometric RDE

$$\begin{aligned} \mathrm {d}{\widetilde{X}}= b({\widetilde{X}}) \diamond \mathrm {d}W , \qquad {\widetilde{X}}(0) = \xi \end{aligned}$$

admits a unique solution ${\widetilde{X}}\in D^{p\text {-}\mathrm {var}}([0,1],{\mathbb {R}}^m)$.

Suppose now that *W* has finitely many jumps at times $0< t_1< \cdots < t_n \le 1$. Then the solution *X* of the forward RDE

$$\begin{aligned} \mathrm {d}X = b(X)^- \mathrm {d}W , \qquad X(0) = \xi \end{aligned}$$

can be obtained by solving the canonical RDE on each of the intervals $[0,t_1), [t_1,t_2),\ldots [t_n, 1)$ (on which *W* is continuous), and requiring that at the jump times

3.6 $$\begin{aligned} X(t_k) = X(t_k-) + b(X(t_k-)) (W(t_k) - W(t_k-)) . \end{aligned}$$

Hence in the case that *W* has finitely many jumps, it is straightforward to construct the solution *X* first on $$[0, t_1)$$, then at $$t_1$$, then on $$[t_1, t_2)$$ and so on. As we shall see, this construction furthermore allows for an easy extension of stability results of continuous RDEs to the setting with jumps.

#### Remark 3.3

The construction of the forward solution for processes with infinitely many discontinuities is more involved, and can be achieved by solving directly the integral equation (3.4). This is done in [15] but is not required here.

Recall that $\phi _b$ is the path function on ${\mathbb {R}}^{1+d+m}$ as in Definition 2.5 with $\ell _{d}$ replaced by $\ell _{1+d}$.

#### Theorem 3.4

Suppose that $\{W_n\}_{n \ge 1}$ is a sequence of $D^{(q,p)\text {-}\mathrm {var}}([0,1], {\mathbb {R}}^{1+d})$-valued random elements with almost surely finitely many jumps. Suppose that $b\in C^{\beta ,\gamma }$. Let $X_n$ be the solution of the forward RDE

$$\begin{aligned} \mathrm {d}X_n = b(X_n)^- \mathrm {d}W_n , \qquad X_n(0) = \xi _n \in {\mathbb {R}}^m . \end{aligned}$$

Suppose that

- $$\lim _{n\rightarrow \infty } \xi _n = \xi $$ for some $$\xi \in {\mathbb {R}}^m$$,
- $W_n\rightarrow _w W$ in $D([0,1],{\mathbb {R}}^{1+d})$ with the ${\mathcal {SM}}_1$ topology as $n \rightarrow \infty$ (we allow the limit process *W* to have infinitely many jumps),
- the family of random variables $\Vert W_n\Vert _{(q,p)\text {-}\mathrm {var}}$ is tight,
- $\sum _t |W_n(t) - W_n(t-)\bigr |^2 \rightarrow _w 0$ as $n \rightarrow \infty$, where the sum is over all jump times of $W_n$.

Then $\Vert W\Vert _{(q,p)\text {-}\mathrm {var}} < \infty$ almost surely. Let *X* be the solution of the geometric RDE

$$\begin{aligned} \mathrm {d}X = b(X) \diamond \mathrm {d}W , \qquad X(0) = \xi . \end{aligned}$$

(The RDE is well-posed because $\Vert W\Vert _{(q,p)\text {-}\mathrm {var}} < \infty$.) Then for each $q'>q$ and $p'>p$,

$$\begin{aligned} ((W_n, X_n), \ell _{1+d+m}) \rightarrow _w ((W, X), \phi _b) \qquad \text {in} \qquad \bigl ({\mathscr {D}}([0,1],{\mathbb {R}}^{1+d+m}), {\varvec{\alpha }}_{(q',p')\text {-}\mathrm {var}}\bigr ) \end{aligned}$$

as $$n\rightarrow \infty $$.

We give the proof after several preliminary results. We will see that if $X_n$ solved the geometric RDE $\mathrm {d}X_n = b(X_n) \diamond \mathrm {d}W_n$ instead of the forward RDE, then Theorem 3.4 would readily follow from [7] (and assumption (d) would not be needed). In Lemma 3.6, we verify that under assumption (d) the solution of the forward RDE $\mathrm {d}X_n = b(X_n)^- \mathrm {d}W_n$ closely approximates the solution of the geometric RDE $\mathrm {d}X_n = b(X_n) \diamond \mathrm {d}W_n$ (generalising a result of [44]). First we show how a single jump of a geometric solution relates to a “forward” jump (cf. [44, Lemma 1.1, Eq. (11)]). Define the semi-norm

$$\begin{aligned} \Vert b\Vert _{{{\,\mathrm{Lip}\,}}} = \sup _{x,y\in {\mathbb {R}}^m} \frac{|b(x)-b(y)|}{|x-y|} . \end{aligned}$$

#### Lemma 3.5

Suppose that $X \in C([0,1], {\mathbb {R}}^m)$ solves the ODE $\mathrm {d}X = b(X) \mathrm {d}t$ with *b* Lipschitz. Then $\bigl |X(1) - X(0) - b(X(0))\bigr | \le \Vert b\Vert _{{{\,\mathrm{Lip}\,}}}\Vert b\Vert _\infty / 2$.

#### Proof

Write $$X(1) = X(0) + b(X(0)) + \int _0^1 \bigl (b(X(t)) - b(X(0))\bigr ) \mathrm {d}t$$. Since $$|X(t) - X(0)| \le \Vert b\Vert _\infty t$$,

$$\begin{aligned} \Bigl | \int _0^1 \bigl (b(X(t)) - b(X(0))\bigr ) \mathrm {d}t \Bigr | \le \Vert b\Vert _{{{\,\mathrm{Lip}\,}}} \int _0^1 |X(t) - X(0)| \mathrm {d}t \le \Vert b\Vert _{{{\,\mathrm{Lip}\,}}}\Vert b\Vert _{\infty } \int _0^1 \, t \mathrm {d}t . \end{aligned}$$

$$\square $$

We now quantify the error in moving from forward to geometric solutions.

#### Lemma 3.6

Suppose that $W \in D^{(q,p)}([0,1], {\mathbb {R}}^{1+d})$ has finitely many jumps. Let $b \in C^{\beta ,\gamma }$ and let $X, {\widetilde{X}}\in D([0,1], {\mathbb {R}}^m)$ be given by

$$\begin{aligned} \mathrm {d}X = b(X)^- \mathrm {d}W, \qquad \mathrm {d}{\widetilde{X}}= b({\widetilde{X}}) \diamond \mathrm {d}W, \qquad X(0)={\widetilde{X}}(0) = \xi . \end{aligned}$$

Then

$$\begin{aligned} \Vert X - {\widetilde{X}}\Vert _{p\text {-}\mathrm {var}} \le \Vert b\Vert _{{{\,\mathrm{Lip}\,}}}\Vert b\Vert _\infty K \sum _{t} |W(t) - W(t-)|^2 , \end{aligned}$$

where *K* depends only on $\Vert b\Vert _{C^{\beta ,\gamma }}$, $\Vert W\Vert _{(q,p)\text {-}\mathrm {var}}$, $\gamma$, $\beta$, *p*, and *q*, and the sum is over all jump times *t* of *W*.

#### Proof

Let $t_1< \cdots < t_n$ be the jump times of *W*; let $t_0 = 0$.

For $j \le n$, define $X_j$ as the solution of forward RDE $\mathrm {d}X_j = b(X_j)^- \mathrm {d}W$, $X_j(0) = \xi$, on $[0, t_j]$, and as the solution of the geometric RDE $\mathrm {d}X_j = b(X_j) \diamond \mathrm {d}W$ on $[t_j, 1]$ with the initial condition taken from the solution on $[0, t_j]$.

For each *j*, the processes $$X_{j-1}$$ and $$X_j$$ coincide on $$[0, t_j)$$ but possibly differ at $$t_j$$. By Lemma 3.5 and the identity (3.6),

3.7 $$\begin{aligned} |X_j(t_j) - X_{j-1}(t_j)| \le \frac{1}{2} \Vert b\Vert _{{{\,\mathrm{Lip}\,}}}\Vert b\Vert _\infty |W(t_j) - W(t_j-)|^2 . \end{aligned}$$

On $[t_j, 1]$, both $X_{n, j-1}$ and $X_{n, j}$ solve the geometric RDE $\mathrm {d}X = b(X) \diamond \mathrm {d}W$, although with possibly different initial conditions. Recall that solutions of geometric RDEs are obtained from RDEs driven by continuous paths by inserting fictitious time intervals and linearly bridging the jumps. As such, they enjoy Lipschitz dependence on the initial condition (see [14, Theorem 12.10])3.8where *K* depends only on $\Vert b\Vert _{C^{\beta ,\gamma }}$, $\Vert W\Vert _{(q,p)\text {-}\mathrm {var}}$, $\gamma$, $\beta$, *p*, and *q*.

It follows from (3.7) and (3.8) thatObserving that $X_0 = {\widetilde{X}}$ and $X_n = X$, and taking the sum over *j*, we obtain the result. $\square$

#### Proof of Theorem 3.4

Denote by ${\varvec{\alpha }}_{(q,p)\text {-}\mathrm {var}}$ the metric on $D^{(q,p)\text {-}\mathrm {var}}([0,1],{\mathbb {R}}^k)$ induced by the corresponding metric on ${\mathscr {D}}^{(q,p)\text {-}\mathrm {var}}([0,1],{\mathbb {R}}^k)$ upon pairing paths with the linear path function $\ell _{k}$, i.e. ${\varvec{\alpha }}_{(q,p)\text {-}\mathrm {var}}(X_1,X_2) = {\varvec{\alpha }}_{(q,p)\text {-}\mathrm {var}}((X_1, \ell _{k}), (X_2, \ell _{k}))$. Let $D^{0,(q,p)\text {-}\mathrm {var}} \subset D^{(q,p)\text {-}\mathrm {var}}$ denote the closure of smooth paths in $(D^{(q,p)\text {-}\mathrm {var}}, {\varvec{\alpha }}_{(q,p)\text {-}\mathrm {var}})$. By the same argument as [7, Proposition 3.10 (v)], note that $D^{(q,p)\text {-}\mathrm {var}} \subset D^{0,(q',p')\text {-}\mathrm {var}}$ for all $q' > q$ and $p' > p$.

Fix $1\le q' \le p'<2$ with $p' \in (p,\gamma )$, $q'\in (q,\beta )$, and such that (3.1) holds with *q*, *p* replaced by $q',p'$. By Chevyrev and Friz [7, Proposition 2.9], convergence in ${\mathcal {SM}}_1$ is equivalent to convergence in $(D, {\varvec{\alpha }}_\infty )$. By the Skorokhod representation theorem, we can thus suppose that a.s. $\lim _{n\rightarrow \infty }{\varvec{\alpha }}_\infty (W_n, W)=0$. Tightness of $\{\Vert W_n\Vert _{(q,p)\text {-}\mathrm {var}}\}$ implies that a.s. there is a subsequence $n_k$ such that $\limsup _{k \rightarrow \infty } \Vert W_{n_k}\Vert _{(q,p)\text {-}\mathrm {var}} < \infty$, and thus $\Vert W\Vert _{(q,p)\text {-}\mathrm {var}}<\infty$ a.s. by lower semi-continuity of (*q*, *p*)-variation. In addition, by a standard interpolation argument (cf. [7, Lemma 3.11]), it holds that ${\varvec{\alpha }}_{(q',p')\text {-}\mathrm {var}}(W_n,W) \rightarrow 0$ in probability, and therefore $W_n \rightarrow _w W$ in $(D^{0,(q',p')\text {-}\mathrm {var}}, {\varvec{\alpha }}_{(q',p')\text {-}\mathrm {var}})$.

Since $(D^{0,(q',p')\text {-}\mathrm {var}}, {\varvec{\alpha }}_{(q',p')\text {-}\mathrm {var}})$ is separable, we can again apply the Skorokhod representation theorem and suppose henceforth that, a.s., $W_n \rightarrow W$ in ${\varvec{\alpha }}_{(q',p')\text {-}\mathrm {var}}$ and $\sum |W_n(t) - W_n(t-)|^2 \rightarrow 0$ (we used here that $\sum |W_n(t) - W_n(t-)|^2$ converges in law to a constant).

An application of the continuity of solution map for generalised geometric RDEs (the proof of [7, Theorem 3.13] combined with [14, Theorem 12.10]; see Remark 3.2) shows that

3.9 $$\begin{aligned} ((W_n, {\widetilde{X}}_n), \phi _b) \rightarrow ((W, X), \phi _b) \; \text { in } \; ({\mathscr {D}}^{(q',p')\text {-}\mathrm {var}}([0,1],{\mathbb {R}}^{1+d+m}), {\varvec{\alpha }}_{(q',p')\text {-}\mathrm {var}}), \end{aligned}$$

where ${\widetilde{X}}_n$ solves the geometric RDE

$$\begin{aligned} \mathrm {d}{\widetilde{X}}_n = b({\widetilde{X}}_n) \diamond \mathrm {d}W_n , \qquad {\widetilde{X}}_n(0) = \xi _n . \end{aligned}$$

Furthermore, since clearly

3.10 $$\begin{aligned} \lim _{n\rightarrow \infty }{\varvec{\alpha }}_\infty (((W_n,{\widetilde{X}}_n),\phi _b),((W_n,{\widetilde{X}}_n),\ell _{1+d+m})) = 0, \end{aligned}$$

it follows from [7, Lemma 3.11] that

3.11 $$\begin{aligned} \lim _{n \rightarrow \infty } {\varvec{\alpha }}_{(q',p')\text {-}\mathrm {var}}(((W_n,{\widetilde{X}}_n),\phi _b),((W_n,{\widetilde{X}}_n),\ell _{1+d+m})) = 0 . \end{aligned}$$

It follows from Lemma 3.6 that $\lim _{n\rightarrow \infty }\Vert (W_n,{\widetilde{X}}_n)-(W_n,X_n)\Vert _{p'\text {-}\mathrm {var}} = 0$, and in particular that ${\varvec{\sigma }}_\infty ((W_n,{\widetilde{X}}_n),(W_n,X_n)) \rightarrow 0$. By virtue of interpolation, for each $q''>q'$ and $p''>p'$, the identity map

$$\begin{aligned} (W,X) \mapsto ((W,X),\ell _{1+d+m}) , \qquad \big (D^{(q',p')\text {-}\mathrm {var}},{\varvec{\sigma }}_\infty \big ) \rightarrow \big (D^{(q',p')\text {-}\mathrm {var}},{\varvec{\alpha }}_{(q'',p'')\text {-}\mathrm {var}}\big ) \end{aligned}$$

is uniformly continuous on sets bounded in $(q',p')$-variation (cf. [7, Proposition 3.12]), from which it follows that

3.12 $$\begin{aligned} \lim _{n\rightarrow \infty }{\varvec{\alpha }}_{(q'',p'')\text {-}\mathrm {var}}(((W_n,{\widetilde{X}}_n),\ell _{1+d+m}),((W_n,X_n),\ell _{1+d+m})) = 0 . \end{aligned}$$

Combining (3.9), (3.11), and (3.12), we obtain

$$\begin{aligned} \lim _{n\rightarrow \infty } {\varvec{\alpha }}_{(q'',p'')\text {-}\mathrm {var}}(((W_n,X_n),\ell _{1+d+m}, ((W,X),\phi _b)) = 0 . \end{aligned}$$

Since $q''>q'>q$ and $p''>p'>p$ are arbitrary, the conclusion follows. $\square$

We are now ready for the proof of Theorem 2.6.

#### Proof of Theorem 2.6

Defining the process $V_n :[0,\infty ) \rightarrow [0,\infty )$, $V_n(t)=n^{-1}\lfloor tn \rfloor$, observe that $X_n$ solves the forward RDE

$$\begin{aligned} \mathrm {d}X_n = a(X_n)^- \mathrm {d}V_n + b(X_n)^- \mathrm {d}W_n . \end{aligned}$$

It follows from our assumptions that

3.13 $$\begin{aligned} (V_n,W_n) \rightarrow ({\mathrm {id}},L) \quad \text {in the }{\mathcal {SM}}_1\text { topology} \end{aligned}$$

and

3.14 $$\begin{aligned} \{\Vert (V_n,W_n)\Vert _{(1,p)\text {-}\mathrm {var}}\}_{n \ge 1} \quad \text {is tight for every }p > \alpha ' . \end{aligned}$$

Furthermore, since $\alpha < 2$ and $W_n$ makes at most *n* jumps of size at most $n^{-1/\alpha } \Vert v\Vert _\infty$,

3.15 $$\begin{aligned} \sum _t |W_n(t) - W_n(t-)\bigr |^2 \le \Vert v\Vert _\infty ^2 n^{1-2/\alpha } \rightarrow 0 \quad \text {as } n \rightarrow \infty . \end{aligned}$$

Choose $p \in (\alpha ',\gamma )$ and $q \in (1, \min \{p, \beta \})$ such that (3.1) is satisfied. By Theorem 3.4, it follows from (3.13), (3.14), and (3.15) that $\Vert L\Vert _{p\text {-}\mathrm {var}}<\infty$ a.s. and

3.16 $$\begin{aligned} ((V_n,W_n, X_n), \ell _{1+d+m}) \rightarrow _w (({\mathrm {id}},L, X),\phi _{(a,b)}) \end{aligned}$$

in $({\mathscr {D}}^{(q,p)\text {-}\mathrm {var}}([0,1],{\mathbb {R}}^{1+d+m}), {\varvec{\alpha }}_{(q,p)\text {-}\mathrm {var}})$. Moreover, $\lim _{n\rightarrow 0}\Vert V_n - {\mathrm {id}}\Vert _{q\text {-}\mathrm {var}} =0$ and thus (3.16) readily implies that $((W_n, X_n), \ell _{d+m}) \rightarrow _w ((L, X),\phi _{b})$ in $({\mathscr {D}}^{p\text {-}\mathrm {var}}([0,1],{\mathbb {R}}^{d +m}), {\varvec{\alpha }}_{p\text {-}\mathrm {var}})$. $\square$

## Results for Gibbs–Markov maps

In this section, we prove results on weak convergence to a Lévy process, and tightness in *p*-variation, for a class of uniformly expanding maps known as Gibbs–Markov maps [1]. The weak convergence result extends work of [1, 22, 33, 42] from scalar-valued observables to ${\mathbb {R}}^d$-valued observables. The result on tightness in *p*-variation is new even for $d=1$.

### Gibbs–Markov maps

Let (*Z*, *d*) be a bounded metric space with Borel sigma-algebra ${\mathcal {B}}$ and finite Borel measure $\nu$, and an at most countable partition ${\mathcal {P}}$ of *Z* (up to a zero measure set) with $\nu (a) > 0$ for each $a \in {\mathcal {P}}$. Let $F:Z\rightarrow Z$ be a nonsingular ergodic measurable transformation. We assume that *F* is a *Gibbs–Markov map*. That is, there are constants $\lambda > 1$, $K > 0$ and $\theta \in (0,1]$ such that for all $z,z'\in a$ and $a \in {\mathcal {P}}$:

- *Fa* is a union of partition elements and *F* restricts to a (measure-theoretic) bijection from *a* to *Fa*; moreover $\inf _{a\in {\mathcal {P}}} \nu (Fa)>0$;
- $$d(Fz, Fz') \ge \lambda d(z,z')$$;
- the inverse Jacobian $\zeta _a = \frac{d\nu }{d\nu \circ F}$ of the restriction $F :a \rightarrow Fa$ satisfies
  4.1 $$\begin{aligned} \bigl | \log \zeta _a(z) - \log \zeta _a(z') \bigr | \le K d(Fz, Fz')^\theta . \end{aligned}$$

It is standard (see for example [1, Corollary p. 199]) that there is a unique *F*-invariant probability measure $\mu _Z$ absolutely continuous with respect to $\nu$, with bounded density $d\mu _Z / d\nu$. The measure $\mu _Z$ is ergodic and we suppose for simplicity that $\mu _Z$ is mixing. (The nonmixing case is also covered by standard arguments, see for example the end of the proof of [33, Proposition 4.3], but is not required here.)

#### Definition 4.1

We say that an ${\mathbb {R}}^d$-valued random variable $\xi$ is *regularly varying* with index $\alpha > 0$ if there exists a probability measure $\sigma$ on ${\mathcal {B}}({\mathbb {S}}^{d-1})$, the Borel sigma-algebra on the unit sphere ${\mathbb {S}}^{d-1} = \{x \in {\mathbb {R}}^d : |x| = 1 \}$, such that

$$\begin{aligned} \lim _{t \rightarrow \infty } \frac{{{\,\mathrm{\mathbb {P}}\,}}(|\xi |> rt, \ \xi / |\xi | \in B)}{{{\,\mathrm{\mathbb {P}}\,}}(|\xi | > t)} = r^{-\alpha } \sigma (B) \end{aligned}$$

for all $r > 0$ and $B \in {\mathcal {B}}({\mathbb {S}}^{d-1})$ with $\sigma (\partial B) = 0$.

Recall that an $\alpha$-stable random variable *X* in ${\mathbb {R}}^d$ with $\alpha \in (1,2)$ and ${{\,\mathrm{\mathbb {E}}\,}}X = 0$ has characteristic function

$$\begin{aligned} {{\,\mathrm{\mathbb {E}}\,}}\exp ( i u \cdot X) = \exp \biggl \{ - \int _{{\mathbb {S}}^{d-1}} |u \cdot s|^\alpha \Bigl ( 1 - i {{\,\mathrm{sgn}\,}}(u \cdot s) \tan \frac{\pi \alpha }{ 2} \Bigr ) \mathrm {d}\Lambda (s) \biggr \} ,\quad u \in {\mathbb {R}}^d . \end{aligned}$$

Here $\Lambda$ is a finite nonnegative Borel measure on ${\mathbb {S}}^{d-1}$ with $\Lambda ({\mathbb {S}}^{d-1})>0$, known as the *spectral measure* [39, Section 2.3]. It is a direct verification that $\gamma X$, with $\gamma \ge 0$, has spectral measure $\gamma ^\alpha \Lambda$.

We say that an $\alpha$-stable Lévy process $L_\alpha$ has spectral measure $\Lambda$ if $L_\alpha (1)$ has spectral measure $\Lambda$.

Fix a function $\tau :Z\rightarrow \{1,2,\ldots \}$ that is constant on each $a\in {\mathcal {P}}$ with value $\tau (a)$ such that $\int _Z \tau \mathrm {d}\mu _Z < \infty$. Let $V:Z\rightarrow {\mathbb {R}}^d$ be integrable with $\int _Z V \mathrm {d}\mu _Z = 0$. Assume that there exists $C_0>0$ such that for and all $z,z' \in a$, $a \in {\mathcal {P}}$,

4.2 $$\begin{aligned} |V(z)| \le C_0\tau (a) \qquad \text {and}\qquad |V(z) - V(z')| \le C_0 \tau (a)d(Fz,Fz')^\theta . \end{aligned}$$

Suppose that $b_n$ is a sequence of positive numbers and define the càdlàg process

$$\begin{aligned} W_n(t) = b_n^{-1} \sum _{j=0}^{\lfloor nt \rfloor -1} V \circ F^j . \end{aligned}$$

We consider $W_n$ as a random element on the probability space $(Z, \mu _Z)$. Throughout this section, $\Vert \cdot \Vert _p$ denotes the $L^p$ norm on $(Z,\mu _Z)$ for $1 \le p \le \infty$ and ${{\,\mathrm{\mathbb {E}}\,}}$ denotes expectation with respect to $\mu _Z$.

We now state the main results of this section.

#### Theorem 4.2

Suppose that

- *V* is regularly varying on $(Z, \mu _Z)$ with index $\alpha \in (1,2)$ and $\sigma$ as in Definition 4.1,
- $$b_n$$ satisfies $$\lim _{n \rightarrow \infty } n \mu _Z( |V| > b_n ) = 1$$,
- $$V - {{\,\mathrm{\mathbb {E}}\,}}(V \mid {\mathcal {P}}) \in L^p$$ for some $$p > \alpha $$.

Then $W_n \rightarrow _w L_\alpha$ in the ${\mathcal {SJ}}_1$ topology as $n \rightarrow \infty$, where $L_\alpha$ is the $\alpha$-stable Lévy process with spectral measure $\Lambda = \cos \frac{\pi \alpha }{2} \Gamma (1-\alpha ) \sigma$.

#### Remark 4.3

- If *V* is regularly varying and $\lim _{n \rightarrow \infty } n \mu _Z( |V| > b_n ) = 1$, then $b_n$ is a regularly varying sequence. In particular, if $\mu _Z( |V| > n) \sim c n^{-\alpha }$ for some $c > 0$, then $b_n \sim c^{1/\alpha } n^{1/\alpha }$.
- In many examples (including the intermittent maps in Sect. 6.2), $\tau \in L^q$ for each $q < \alpha$, and there exist $C > 0$ and $\beta \in (0,1)$ such that $|V(z) - V(z')| \le C \tau ^\beta$ for all $z, z' \in a$, $a \in {\mathcal {P}}$. This implies that $V - {{\,\mathrm{\mathbb {E}}\,}}(V \mid {\mathcal {P}}) \in L^p$ for some $p > \alpha$.

#### Theorem 4.4

Suppose that $\tau$ is regularly varying with index $\alpha \in (1,2)$ on $(Z, \mu _Z)$, and that $b_n$ satisfies $\lim _{n\rightarrow \infty } n\mu _Z(\tau >b_n)=1$. Then $\sup _n\int _Z \Vert W_n\Vert _{p\text {-}\mathrm {var}} \mathrm {d}\mu _Z < \infty$ for all $p>\alpha$.

### Preliminaries about Gibbs–Markov maps

We recall the following standard result.

#### Lemma 4.5

Let $V :Z\rightarrow {\mathbb {R}}^d$ be integrable with $\int _Z V \mathrm {d}\mu _Z = 0$ and satisfying (4.2). Then

- $V=m+\chi \circ F - \chi$, where *m* is integrable with ${{\,\mathrm{\mathbb {E}}\,}}(m \mid F^{-1}{\mathcal {B}})=0$, and $\Vert \chi \Vert _\infty \le C C_0$ with $C > 0$ independent of *V*.
- For every $p \in (1,2]$ there is a constant *C*(*p*), depending only on *p*, such that
  $$\begin{aligned} \biggl \Vert \max _{k \le n} \Bigl | \sum _{j=0}^{k-1} V \circ F^j \Bigr | \biggr \Vert _p \le C(p) n^{1/p} (\Vert \chi \Vert _\infty + \Vert V\Vert _p) . \end{aligned}$$
  (We do not exclude the case $\Vert V\Vert _p = \infty$.)

#### Proof

For $z, z' \in Z$, let $s(z, z')$ be the *separation time*, i.e. the minimal nonnegative integer such that $F^{s(z, z')}(z)$ and $F^{s(z, z')}(z')$ belong to different elements of ${\mathcal {P}}$. Let $d_\theta$ be the separation metric on *Z*:

$$\begin{aligned} d_\theta (z, z') = \lambda ^{- \theta s(z, z')} . \end{aligned}$$

Note that $d(z, z')^\theta \le d_\theta (z, z') ({{\,\mathrm{diam}\,}}Z)^\theta$, so $\theta$-Hölder observables with respect to *d* are $d_\theta$-Lipschitz. For an observable $\phi :Z \rightarrow {\mathbb {R}}^d$, let

$$\begin{aligned} \Vert \phi \Vert = \Vert \phi \Vert _\infty + \sup _{z \ne z' } \frac{|\phi (z) - \phi (z')|}{d_\theta (z,z')} . \end{aligned}$$

Let $P :L^1(\mu _Z) \rightarrow L^1(\mu _Z)$ be the transfer operator corresponding to *F* and $\mu _Z$, i.e. $\int _Z P \phi \,w \mathrm {d}\mu _Z = \int _Z \phi \,w\circ F \mathrm {d}\mu _Z$ for all $\phi \in L^1$, $w\in L^\infty$.

By for example [1, Section 1], there are constants $C_1>0$, $\gamma \in (0,1)$ such that $\Vert P^k \phi \Vert \le C_1 \gamma ^k \Vert \phi \Vert$ for all $\phi :Z \rightarrow {\mathbb {R}}^d$ with ${{\,\mathrm{\mathbb {E}}\,}}\phi = 0$ and all $k \ge 0$.

By Melbourne and Nicol [30, Lemma 2.2], there is a constant $C_2>0$ independent of *V* such that $\Vert PV\Vert \le C_0 C_2$ for all *V* satisfying the stated conditions. Hence

$$\begin{aligned} \Vert P^k V\Vert = \Vert P^{k-1}P V\Vert \le C_1 \gamma ^{k-1}\Vert PV\Vert \le C_0 C_1 C_2 \gamma ^{k-1} . \end{aligned}$$

Let $\chi = \sum _{k=1}^\infty P^k V$. Then $\Vert \chi \Vert _\infty \le \Vert \chi \Vert \le C_0C_1C_2(1-\gamma )^{-1}$. Let $m = V - \chi \circ F + \chi$. Define $U :L^1(\mu _Z)\rightarrow L^1(\mu _Z)$ by $U\phi =\phi \circ F$. Then $PU=I$ and $UP={{\,\mathrm{\mathbb {E}}\,}}(\,\cdot \mid F^{-1}{\mathcal {B}})$. Hence ${{\,\mathrm{\mathbb {E}}\,}}(m\mid F^{-1}{\mathcal {B}})=UPm=U(PV-\chi +P\chi )=0$ proving part (a).

For part (b), we proceed as in the proof of [33, Proposition 4.3]. Fix $n > 0$ and let $M^n_k = \sum _{j=n-k}^{n-1} m \circ F^j$. By (a), $M^n_k$ is a martingale on $0 \le k \le n$. By Burkholder’s inequality, there is a constant *C*(*p*) depending only on *p* such that

$$\begin{aligned} \bigl \Vert \max _{k \le n} |M^n_k| \bigr \Vert _p \le C(p) n^{1/p} \Vert m\Vert _p \le C(p) n^{1/p} ( 2 \Vert \chi \Vert _\infty + \Vert V\Vert _p) . \end{aligned}$$

Next,

$$\begin{aligned} \biggl \Vert \max _{k \le n} \Bigl | \sum _{j=0}^{k-1} V \circ F^j \Bigr | \biggr \Vert _p \le 2 \Vert \chi \Vert _\infty + 2 \bigl \Vert \max _{k \le n} |M^n_k| \bigr \Vert _p , \end{aligned}$$

and part (b) follows. $\square$

For sigma-algebras ${\mathcal {F}}$ and ${\mathcal {G}}$ on a common probability space $(\Omega ,{{\,\mathrm{\mathbb {P}}\,}})$, define

$$\begin{aligned} \psi ({\mathcal {F}}, {\mathcal {G}}) = \sup \biggl \{ \frac{\bigl |{{\,\mathrm{\mathbb {P}}\,}}(A \cap B) - {{\,\mathrm{\mathbb {P}}\,}}(A) {{\,\mathrm{\mathbb {P}}\,}}(B) \bigr |}{{{\,\mathrm{\mathbb {P}}\,}}(A){{\,\mathrm{\mathbb {P}}\,}}(B)} : A \in {\mathcal {F}}, \ B \in {\mathcal {G}}\biggr \} . \end{aligned}$$

For $0\le n \le k$, let ${\mathcal {P}}_n^k$ be the smallest sigma-algebra which contains $F^{-j} {\mathcal {P}}$ for $j=n,\ldots , k$. A standard property of mixing Gibbs–Markov maps (see for example [1, Section 1]) is that there exist $\gamma \in (0,1)$ and $C>0$ such that for all $k \ge 0$, $n \ge 1$,

4.3 $$\begin{aligned} \psi \big ({\mathcal {P}}_0^k, {\mathcal {P}}_{n+k}^\infty \big ) \le C\gamma ^n, \end{aligned}$$

where the probability measure in the definition of $\psi$ is $\mu _Z$.

### Weak convergence to a Lévy process

In this subsection, we prove Theorem 4.2. We use the following result due to Tyran-Kamińska [41].

#### Theorem 4.6

Let $X_0, X_1, \ldots$ be a strictly stationary sequence of integrable ${\mathbb {R}}^d$-valued random variables with ${{\,\mathrm{\mathbb {E}}\,}}X_0 = 0$. For $0\le n \le k$, let ${\mathcal {F}}_n^k$ denote the sigma-algebra generated by $\{X_n, \ldots , X_k\}$. Suppose that:

- $X_0$ is regularly varying with index $\alpha \in [1,2)$ and $\sigma$ as in Definition 4.1.
- $$\sum _{j \ge 0} \psi (2^j) < \infty $$, where $$\psi (n) = \sup _{k\ge 0} \psi ({\mathcal {F}}_0^k, {\mathcal {F}}_{n+k}^\infty )$$.
- $\lim _{n \rightarrow \infty } {{\,\mathrm{\mathbb {P}}\,}}\bigl ( |X_j|> \epsilon b_n \ \big \vert \ |X_0| > \epsilon b_n \bigr ) = 0$ for all $\epsilon > 0$ and $j \ge 1$, where the sequence $b_n$ is such that $\lim _{n \rightarrow \infty } n {{\,\mathrm{\mathbb {P}}\,}}(|X_0| > b_n) = 1$.

Then as $n \rightarrow \infty$, the random process $W_n$ given by $W_n(t) = b_n^{-1} \sum _{j = 0}^{\lfloor nt \rfloor -1} X_j$ converges to an $\alpha$-stable Lévy process $L_\alpha$ in $D([0,1], {\mathbb {R}}^d)$ in the ${\mathcal {SJ}}_1$ topology.

#### Remark 4.7

It is implicit in [41] that $L_\alpha$ has spectral measure $\Lambda = \cos \frac{\pi \alpha }{2} \Gamma (1 - \alpha ) \sigma$, where $\sigma$ is the measure on ${\mathbb {S}}^{d-1}$ for $X_0$ as in Definition 4.1.

#### Proof of Theorem 4.6

We verify the hypotheses of [41, Theorem 1.1]. In the notation of [41], observe that (b) and [41, Lemma 4.8] together with $\rho \le \psi$ imply that [41, Eq. (1.6)] holds. Moreover, (c) and [41, Corollary 1.3] together with $\varphi \le \psi$ imply that [41, **LD**($\phi _0$)] holds (for inequalities concerning $\rho$, $\psi$, and $\varphi$, see [4]). $\square$

Write $V = V' + V''$ where $V' = {{\,\mathrm{\mathbb {E}}\,}}(V \mid {\mathcal {P}})$. Let

$$\begin{aligned} W'_n(t) = b_n^{-1} \sum _{j=0}^{\lfloor nt \rfloor -1} V' \circ F^j , \qquad W''_n(t) = b_n^{-1} \sum _{j=0}^{\lfloor nt \rfloor -1} V'' \circ F^j . \end{aligned}$$

#### Proposition 4.8

(i) $W'_n$ converges in ${\mathcal {SJ}}_1$ to the $\alpha$-stable Lévy process $L_\alpha$ with spectral measure $\Lambda = \cos \frac{\pi \alpha }{2} \Gamma (1 - \alpha ) \sigma$.
(ii) $$\bigl \Vert \sup _{t\in [0,1]}|W''_n(t)|\bigr \Vert _1 \rightarrow 0$$ as $$n \rightarrow \infty $$.

#### Proof

To prove part (i), we verify the hypotheses of Theorem 4.6 with $X_k = V' \circ F^k$. Since $\mu _Z$ is *F*-invariant, $\{V' \circ F^k\}_{k \ge 0}$ is a strictly stationary sequence of ${\mathbb {R}}^d$-valued random variables. The remaining hypotheses are verified as follows

- The observable *V* is regularly varying with index $\alpha$ and measure $\sigma$, and $V'' \in L^p$ with $p > \alpha$, so $V'=V-V''$ is regularly varying with the same $\alpha$ and $\sigma$.
- This is a consequence of (4.3).
- It follows from (4.3) and invariance of $\mu _Z$ under *F* that
  $$\begin{aligned} \mu _Z \bigl ( |V'\circ F^j|> \epsilon b_n \ \big \vert \ |V'|> \epsilon b_n \bigr ) \lesssim \mu _Z (|V'| > \epsilon b_n) . \end{aligned}$$

Now we prove part (ii). By the assumptions of Theorem 4.2, $V'' \in L^p$ for some $p \in ( \alpha ,2)$. Note that $|V''| \lesssim \tau$, ${{\,\mathrm{\mathbb {E}}\,}}V'' = 0$ and for each $z,z' \in a$, $a \in {\mathcal {P}}$,

$$\begin{aligned} |V''(z) - V''(z')|=|V(z)-V(z')| \le C_0 \tau (a) d(Fz, Fz')^\theta \; . \end{aligned}$$

Hence by Lemma 4.5(b), $\bigl \Vert \max _{k \le n} | \sum _{j=0}^{k-1} V'' \circ F^j | \bigr \Vert _p \lesssim n^{1/p} = o(b_n)$.

#### Proof of Theorem 4.2

By Proposition 4.8, $$W_n=W_n'+W_n'' \rightarrow _w L_\alpha $$. $$\square $$

### Tightness in *p*-variation

In this subsection we prove Theorem 4.4.

First we record the following elementary properties of $\tau$. (The Gibbs–Markov structure is not required here; the proof only uses that $\tau$ is regularly varying with values in $\{1,2,\ldots \}$ and that $\mu _Z$ is *F*-invariant.)

#### Proposition 4.9

Let $$p>\alpha $$. Then

- $${{\,\mathrm{\mathbb {E}}\,}}(\tau ^p 1_{\{\tau \le b_n\}})=O(n^{-1}b_n^p)$$,
- $${{\,\mathrm{\mathbb {E}}\,}}(\tau 1_{\{\tau \ge b_n\}})=O(n^{-1}b_n)$$,
- $${{\,\mathrm{\mathbb {E}}\,}}\big \{ \big (\sum _{j=0}^{n-1} \tau ^p \circ F^j\big )^{1/p} \big \} =O(b_n)$$.

#### Proof

We have

$$\begin{aligned} {{\,\mathrm{\mathbb {E}}\,}}\big (\tau ^p1_{\{\tau \le b_n\}}\big )= & {} \sum _{j\le b_n}j^p\mu _Z(\tau =j) \le \sum _{j\le b_n}(j^p-(j-1)^p)\mu _Z(\tau \ge j)\\\le & {} p\sum _{j\le b_n}j^{p-1}\mu _Z(\tau \ge j) . \end{aligned}$$

By Karamata’s theorem [3, Proposition 1.5.8], ${{\,\mathrm{\mathbb {E}}\,}}(\tau ^p1_{\{\tau \le b_n\}})\lesssim b_n^p\mu _Z(\tau \ge b_n)$, so part (a) follows by definition of $b_n$. A similar calculation proves part (b). Next,

$$\begin{aligned} \left( \sum _{j=0}^{n-1} \tau ^p \circ F^j\right) ^{1/p}&\le \left( \sum _{j=0}^{n-1} \left( \tau ^p 1_{\{\tau> b_n\}}\right) \circ F^j\right) ^{1/p} + \left( \sum _{j=0}^{n-1} \left( \tau ^p 1_{\{\tau \le b_n\}} \right) \circ F^j\right) ^{1/p}\\&\le \sum _{j=0}^{n-1} \left( \tau 1_{\{\tau > b_n\}}\right) \circ F^j + \left( \sum _{j=0}^{n-1} \left( \tau ^p 1_{\{\tau \le b_n\}} \right) \circ F^j\right) ^{1/p} . \end{aligned}$$

By Jensen’s inequality, invariance of $\mu _Z$ and parts (a) and (b),

$$\begin{aligned}&{{\,\mathrm{\mathbb {E}}\,}}\left\{ \left( \sum _{j=0}^{n-1} \tau ^p \circ F^j\right) ^{1/p} \right\} \\&\quad \le \sum _{j=0}^{n-1} {{\,\mathrm{\mathbb {E}}\,}}\bigl \{ \bigl (\tau 1_{\{\tau> b_n\}}\bigr ) \circ F^j \bigr \} + \left( \sum _{j=0}^{n-1} {{\,\mathrm{\mathbb {E}}\,}}\bigl \{ \bigl (\tau ^p 1_{\{\tau \le b_n\}} \bigr ) \circ F^j \bigr \} \right) ^{1/p}\\&\quad = n {{\,\mathrm{\mathbb {E}}\,}}( \tau 1_{\{\tau > b_n\}}) + \big ( n {{\,\mathrm{\mathbb {E}}\,}}( \tau ^p 1_{\{\tau \le b_n\}}) \big )^{1/p} \lesssim b_n , \end{aligned}$$

proving part (c). $$\square $$

Write $$V=V_n'-{{\,\mathrm{\mathbb {E}}\,}}V_n'+V_n''$$, where

$$\begin{aligned} \textstyle V_n'=V1_{\{\tau > b_n\}}, \quad V_n''=V1_{\{\tau \le b_n\}}-{{\,\mathrm{\mathbb {E}}\,}}( V1_{\{\tau \le b_n\}}) . \end{aligned}$$

Accordingly, define $W_n=W_n'-{{\,\mathrm{\mathbb {E}}\,}}W_n'+W_n''$, where

$$\begin{aligned} W_n'(t)=b_n^{-1}\sum _{j=0}^{\lfloor nt \rfloor -1} V_n'\circ F^j, \qquad W_n''(t)=b_n^{-1}\sum _{j=0}^{\lfloor nt \rfloor -1} V_n''\circ F^j . \end{aligned}$$

#### Proposition 4.10

$$\sup _n{{\,\mathrm{\mathbb {E}}\,}}\Vert W_n'\Vert _{1\text {-}\mathrm {var}} < \infty $$.

#### Proof

By Proposition 4.9(b), ${{\,\mathrm{\mathbb {E}}\,}}|V_n'|\le C_0 {{\,\mathrm{\mathbb {E}}\,}}\bigl (\tau 1_{\{\tau > b_n\}}\bigr ) \lesssim n^{-1}b_n$. Hence

$$\begin{aligned} {{\,\mathrm{\mathbb {E}}\,}}\Vert W_n'\Vert _{1\text {-}\mathrm {var}}={{\,\mathrm{\mathbb {E}}\,}}\left( b_n^{-1}\sum _{j=0}^{n-1}|V_n'|\circ F^j\right) = nb_n^{-1} {{\,\mathrm{\mathbb {E}}\,}}|V_n'|=O(1) , \end{aligned}$$

as required. $$\square $$

#### Proposition 4.11

$$\sup _n{{\,\mathrm{\mathbb {E}}\,}}\Vert W_n''\Vert _{p\text {-}\mathrm {var}}^p < \infty $$ for all $$p\in (\alpha , 2)$$.

#### Proof

Note that ${{\,\mathrm{\mathbb {E}}\,}}V_n''=0$, that $|V_n''|\le |V|+{{\,\mathrm{\mathbb {E}}\,}}|V|\le C_1\tau$ where $C_1=C_0+{{\,\mathrm{\mathbb {E}}\,}}|V|$, and that $|V_n''(z)-V_n''(z')|\le |V(z)-V(z')|\le C_0\tau (a) d(Fz,Fz')^\theta$ for all $z,z'\in a$, $a\in {\mathcal {P}}$. By Lemma 4.5(a), $V''_n = m_n + \chi _n \circ F - \chi _n$, where $\sup _n \Vert \chi _n\Vert _\infty < \infty$ and ${{\,\mathrm{\mathbb {E}}\,}}(m_n \mid F^{-1} {\mathcal {B}}) = 0$. Then

$$\begin{aligned} \Vert m_n\Vert _p \le \Vert V_n''\Vert _p + 2\Vert \chi _n\Vert _p \le 2\Vert V 1_{\{\tau \le b_n\}}\Vert _p + 2\Vert \chi _n\Vert _\infty \end{aligned}$$

and ${{\,\mathrm{\mathbb {E}}\,}}|V1_{\{\tau \le b_n\}}|^p \le C_0^p{{\,\mathrm{\mathbb {E}}\,}}\big ( \tau ^p 1_{\{\tau \le b_n\}}\big ) \lesssim n^{-1}b_n^p$ by Proposition 4.9(a). The assumptions of Theorem 4.4 imply that $b_n^p \gtrsim n$. Hence

4.4 $$\begin{aligned} {{\,\mathrm{\mathbb {E}}\,}}|m_n|^p \lesssim n^{-1}b_n^p . \end{aligned}$$

Write $$W_n''=M_n+B_n$$ where

$$\begin{aligned} M_n(t)= & {} b_n^{-1}\sum _{j=0}^{\lfloor nt \rfloor -1} m_n\circ F^j, \quad B_n(t)=b_n^{-1}\sum _{j=0}^{\lfloor nt \rfloor -1} (\chi _n\circ F-\chi _n)\circ F^j \\= & {} b_n^{-1}(\chi _n\circ F^{\lfloor nt \rfloor }-\chi _n) . \end{aligned}$$

Let $M_n^-(t) = b_n^{-1} \sum _{j=1}^{\lfloor nt \rfloor } m_n \circ F^{n-j} .$ Then $M_n^-$ is a martingale since ${{\,\mathrm{\mathbb {E}}\,}}(m_n \mid F^{-1} {\mathcal {B}}) = 0$. By [36, Theorem 2.1] and (4.4),

4.5 $$\begin{aligned} {{\,\mathrm{\mathbb {E}}\,}}\Vert M_n\Vert _{p\text {-}\mathrm {var}}^p = {{\,\mathrm{\mathbb {E}}\,}}\Vert M_n^-\Vert _{p\text {-}\mathrm {var}}^p \lesssim b_n^{-p} \sum _{j=1}^n {{\,\mathrm{\mathbb {E}}\,}}|m_n \circ F^{n-j}|^p = nb_n^{-p} {{\,\mathrm{\mathbb {E}}\,}}|m_n|^p \lesssim 1 . \end{aligned}$$

Finally, $$\Vert B_n\Vert _{p\text {-}\mathrm {var}}^p \le b_n^{-p}\, n\, (2\Vert \chi _n\Vert _\infty )^p\lesssim nb_n^{-p}\lesssim 1$$ for $$p > \alpha $$.

#### Remark 4.12

For our purposes, it is sufficient to control the first moment ${{\,\mathrm{\mathbb {E}}\,}}\Vert W_n''\Vert _{p\text {-}\mathrm {var}}$. Hence we could have used the simpler result [26, Proposition 2] in place of the sharp result [36, Theorem 2.1]; this would give $\sup _n{{\,\mathrm{\mathbb {E}}\,}}\Vert W_n''\Vert _{p\text {-}\mathrm {var}}^q<\infty$ for all $p > \alpha$ and $q < p$.

#### Proof of Theorem 4.4

Combine Propositions 4.10 and 4.11. $\square$

## Inducing weak convergence and tightness in *p*-variation

A general principle in smooth ergodic theory is that limit laws for dynamical systems are often inherited from the corresponding laws for a suitable induced system [18, 20, 31, 33, 38]. In this section, we show that this principle applies to weak convergence in $D([0,1],{\mathbb {R}}^d)$ with the ${\mathcal {SM}}_1$ topology and to tightness in *p*-variation. The results hold in a purely probabilistic setting.

Let *Y* be a measurable space and $f :Y \rightarrow Y$ a measurable transformation. Suppose that $Z\subset Y$ is a measurable subset with a measurable return time $\tau :Z \rightarrow \{1,2,\ldots \}$, i.e. $f^{\tau (z)}(z) \in Z$ for each $z \in Z$. (It is not assumed that $\tau$ is the first return time.) Define the induced map

$$\begin{aligned} F :Z \rightarrow Z , \qquad Fz = f^{\tau (z)}(z) . \end{aligned}$$

Suppose that $\mu _Z$ is an ergodic *F*-invariant probability measure and that $\bar{\tau }=\int _Z\tau \mathrm {d}\mu _Z < \infty$.

Define the tower $f_\Delta :\Delta \rightarrow \Delta$

5.1 $$\begin{aligned} \Delta = \{ (z, \ell ) : z \in Z, 0 \le \ell< \tau (z) \} , \qquad f_\Delta (z, \ell ) = {\left\{ \begin{array}{ll} (z, \ell + 1), &{} \ell < \tau (z) - 1 , \\ (Fz, 0), &{} \ell = \tau (z) - 1 , \end{array}\right. } \end{aligned}$$

with ergodic $f_\Delta$-invariant probability measure $\mu _\Delta =(\mu _Z \times \mathrm{counting}) / \bar{\tau }$. The map $\pi :\Delta \rightarrow Y$, $\pi (z,\ell )=f^\ell z$ defines a measurable semiconjugacy between $f_\Delta$ and *f*, so $\mu =\pi _*\mu _\Delta$ is an ergodic *f*-invariant probability measure on *Y*.

It is convenient to identify *Z* with $Z \times \{0\} \subset \Delta$. Then on the tower, $\tau$ is the first return time to *Z*.

Let $v :Y \rightarrow {\mathbb {R}}^d$ be measurable and define the corresponding *induced observable*

5.2 $$\begin{aligned} V :Z \rightarrow {\mathbb {R}}^d , \qquad V(z) = \sum _{j=0}^{\tau (z)-1} v(f^jz) . \end{aligned}$$

Let $v_k = \sum _{j=0}^{k-1} v \circ f^j$. To measure how well the excursion $\{v_k(z)\}_{0 \le k \le \tau (z)}$ approximates the straight and monotone path from 0 to *V*(*z*), we define $V^* :Z \rightarrow {\mathbb {R}}^d$,

5.3 $$\begin{aligned} V^* = \inf _{c \in {\mathbb {R}}^d, |c| = 1} \Bigl ( \max _{0 \le k \le \ell \le \tau } c \cdot \bigl (v_k - v_\ell \bigr ) + \max _{0 \le k \le \tau } \bigl | v_k - (c \cdot v_k) c\bigr | \Bigr ) . \end{aligned}$$

Note that $V^*(z) = 0$ if and only if there exist $0 = s_0 \le s_1 \le \cdots \le s_{\tau (z)} = 1$ such that $v_k(z) = s_k V(z)$ for $0 \le k \le \tau (z)$.

Let $b_n$ be a sequence of positive numbers, bounded away from 0, and define

5.4 $$\begin{aligned} W_n(t) = b_n^{-1} \sum _{j=0}^{\lfloor nt \rfloor -1} v \circ f^j \qquad \text {and} \qquad {\widetilde{W}}_n(t) = b_n^{-1} \sum _{j=0}^{\lfloor nt \rfloor -1} V \circ F^j . \end{aligned}$$

In this section, the notation $\rightarrow _\mu$ and $\rightarrow _{\mu _Z}$ is used to denote weak convergence for random variables defined on the probability spaces $(Y, \mu )$ and $(Z, \mu _Z)$ respectively. We prove:

### Theorem 5.1

Suppose that ${\widetilde{W}}_n \rightarrow _{\mu _Z} {\widetilde{W}}$ in the ${\mathcal {SM}}_1$ topology for some random process ${\widetilde{W}}$. Suppose further that

$$\begin{aligned} b_n^{-1} \max _{k<n} V^* \circ F^k \rightarrow _{\mu _Z} 0 . \end{aligned}$$

Then $W_n \rightarrow _{\mu } W$ in the ${\mathcal {SM}}_1$ topology where $W(t) = {\widetilde{W}}(t / \bar{\tau })$.

### Theorem 5.2

Suppose that $\tau$ is regularly varying with index $\alpha >1$ on $(Z, \mu _Z)$, and that $b_n$ satisfies $\lim _{n\rightarrow \infty } n\mu _Z(\tau >b_n)=1$. Let $v\in L^\infty$. Suppose that the family of random variables $\Vert {\widetilde{W}}_n\Vert _{p\text {-}\mathrm {var}}$ is tight on $(Z,\mu _Z)$ for some $p>\alpha$. Then the family $\Vert W_n\Vert _{p\text {-}\mathrm {var}}$ is tight on $(Y, \mu )$.

### Remark 5.3

The assumptions of Theorem 5.2 on $\tau$ can be relaxed. If $\tau ' :Z \rightarrow \{1,2,\ldots \}$ is regularly varying with index $\alpha > 1$ on $(Z, \mu _Z)$ and $b_n$ satisfies $\lim _{n\rightarrow \infty } n\mu _Z(\tau ' > b_n)=1$, then the result holds for all $\tau \le \tau '$.

### Inducing convergence in ${\mathcal {SM}}_1$ topology

In this subsection, we prove Theorem 5.1. Our proof closely follows the analogous proof in [33], with the difference that we work in ${\mathbb {R}}^d$ instead of ${\mathbb {R}}$.

Since $\pi :\Delta \rightarrow Y$ is a measure-preserving semiconjugacy, we may suppose without loss of generality that $Y = \Delta$ and $f=f_\Delta$ as in (5.1). In particular, we may suppose that $\tau$ is the first return time.

Define

$$\begin{aligned} u :Y \rightarrow {\mathbb {R}}^d , \qquad u(y) = {\left\{ \begin{array}{ll} V(z) , &{}\quad y=(z,\tau (z)-1) , \\ 0, &{}\quad \text {otherwise} . \end{array}\right. } \end{aligned}$$

Let

$$\begin{aligned} U_n(t) = b_n^{-1} \sum _{j=0}^{\lfloor nt \rfloor -1} u \circ f^j . \end{aligned}$$

Thus defined, the restriction of $U_n$ to *Z* corresponds to $U_n$ in [33].

#### Lemma 5.4

$U_n \rightarrow _{\mu _Z} W$ in the ${\mathcal {SM}}_1$ topology.

#### Proof

For the case $d=1$, see [33, Lemma 3.4]. The proof for all $d \ge 1$ goes through unchanged. $\square$

Next we control *excursions:* we estimate the distance between $U_n$ and $W_n$ in the ${\mathcal {SM}}_1$ topology.

#### Proposition 5.5

Let $w \in D([T_0,T_1], {\mathbb {R}}^d)$ and define $\phi :[T_0,T_1]\rightarrow {\mathbb {R}}^d$ to be the linear path with $\phi (T_0) = w(T_0)$ and $\phi (T_1) = w(T_1)$. Then for each $c \in {\mathbb {R}}^d$ with $|c| = 1$,

$$\begin{aligned} d_{{\mathcal {SM}}_1}(w, \phi ) \le T_1 - T_0 + 2 \sup _{T_0 \le s < t \le T_1} c \cdot w(t,s) + 2 \sup _{T_0 \le t \le T_1} \bigl | w(T_0, t) - (c \cdot w(T_0, t)) c\bigr | , \end{aligned}$$

where $$w(a,b) = w(b) - w(a)$$.

#### Proof

Without loss of generality, we suppose that $w(T_0) = 0$. Define $\chi :[T_0, T_1] \rightarrow [0,\infty )$ and $\psi :[T_0, T_1]\rightarrow {\mathbb {R}}^d$ to be $\chi (t) = \sup _{s \le t} c \cdot w(s)$ and $\psi (t) = \chi (t) c$. Then $\psi$ is a monotone path in the direction of *c*.

Observe that $|w(t) - \psi (t)| \le \chi (t) - c \cdot w(t) + |w(t) - (c \cdot w(t)) c|$. Hence

5.5 $$\begin{aligned} \sup _t|w(t)-\psi (t)| \le \sup _{s < t} c \cdot w(t,s) + \sup _{t} | w(t) - (c \cdot w(t)) c | . \end{aligned}$$

Further, let $\xi :[T_0, T_1]\rightarrow {\mathbb {R}}^d$ be the linear path with $\xi (T_0) = w(T_0) = 0$ and $\xi (T_1) = \psi (T_1) = \chi (T_1) c$. Since $\xi$ is a reparametrisation of $\psi$ (up to linear jumps),

5.6 $$\begin{aligned} d_{{\mathcal {SM}}_1}(\xi , \psi ) \le T_1 - T_0 . \end{aligned}$$

Also, for each $\epsilon > 0$ there is $s \in [T_0, T_1]$ such that $|\chi (T_1) - c \cdot w(s)| \le \epsilon$. Then

5.7 $$\begin{aligned} \begin{aligned} \sup _t|\phi (t) - \xi (t)|&= |\phi (T_1) - \xi (T_1)| \le |w(T_1) - (c \cdot w(s)) c| + \epsilon \\&\le |w(T_1) - (c \cdot w(T_1)) c | + c \cdot (w(s) - w(T_1)) + \epsilon . \end{aligned} \end{aligned}$$

The result follows from (5.5), (5.6), (5.7) and that $\epsilon$ can be taken arbitrarily small. $\square$

For $s \le t$, let $d_{{\mathcal {SM}}_1, [s,t]}$ denote the distance on [*s*, *t*]. Let $\tau _k = \sum _{j=0}^{k-1} \tau \circ F$.

#### Corollary 5.6

For each *n* and *k*, on *Z*,

$$\begin{aligned} d_{{\mathcal {SM}}_1, [0, \tau _k / n]} (U_n, W_n) \le 2 \max _{0\le j<k} \Bigl \{ \frac{\tau \circ F^j}{n} + \frac{V^* \circ F^j}{b_n} \Bigr \} . \end{aligned}$$

#### Proof

Denote $$T_j = \tau _j / n$$. Since we restrict to *Z*, each interval $$[T_j, T_{j+1}]$$, including with $$j=0$$, corresponds to a complete excursion with $$U_n(T_j) = W_n(T_j)$$ and $$U_n(T_{j+1}) = W_n(T_{j+1})$$. Fix *j* and let $$\phi :[T_j, T_{j+1}]\rightarrow {\mathbb {R}}^d$$ be the linear path such that $$\phi (T_j) = U_n(T_j)$$ and $$\phi (T_{j+1}) = U_n(T_{j+1})$$. Recall that $$U_n$$ is constant on $$[T_j, T_{j+1})$$. By Proposition 5.5,

$$\begin{aligned} d_{{\mathcal {SM}}_1, [T_j, T_{j+1}]}(U_n, \phi )&\le T_{j+1} - T_j , \\ d_{{\mathcal {SM}}_1, [T_j, T_{j+1}]}(W_n, \phi )&\le T_{j+1} - T_j + \frac{2}{b_n} V^* \circ F^j . \end{aligned}$$

Hence

$$\begin{aligned} d_{{\mathcal {SM}}_1, [T_j, T_{j+1}]} (U_n, W_n) \le 2(T_{j+1} - T_j) + \frac{2}{b_n} V^* \circ F^j = \frac{2}{n}\tau \circ F^j + \frac{2}{b_n} V^* \circ F^j . \end{aligned}$$

Finally,

$$\begin{aligned} d_{{\mathcal {SM}}_1, [0,T_k]} (U_n, W_n) \le \max _{j < k} d_{{\mathcal {SM}}_1, [T_j, T_{j+1}]}(U_n, W_n) \; , \end{aligned}$$

and the result follows. $\square$

#### Lemma 5.7

$$d_{{\mathcal {SM}}_1, [0, T]}(U_n, W_n) \rightarrow _{\mu _Z} 0$$ for all $$T > 0$$.

#### Proof

Fix $T > 0$ and define the random variables $k = k(n) = \max \{ j \ge 0: \tau _j / n \le T \}$ on *Z*. Consider the processes $U_n$, $W_n$ on *Z*, where the time interval $[0, \tau _k / n]$ corresponds to *k* complete excursions, while $[\tau _k / n, T]$ is the final incomplete excursion.

By Corollary 5.6 and the assumptions of Theorem 5.1,

$$\begin{aligned} d_{{\mathcal {SM}}_1, [0,\tau _k / n]} (U_n, W_n) \le 2 \max _{j<k} \Bigl \{ \frac{\tau \circ F^j}{n} + \frac{V^* \circ F^j}{b_n} \Bigr \} \rightarrow _{\mu _Z} 0 . \end{aligned}$$

For $y=(z,\ell ) \in Y$, let $E(y) = \sum _{j=0}^{\tau (z) - 1} \bigl |v(f^j z)\bigr |$. Since $\mu$ is *f*-invariant and $b_n \rightarrow \infty$, we have $b_n^{-1} E \circ f^{\lfloor nT \rfloor } \rightarrow _{\mu } 0$. Since $\mu _Z$ is absolutely continuous with respect to $\mu$, we also have $b_n^{-1} E \circ f^{\lfloor nT \rfloor } \rightarrow _{\mu _Z} 0$. Hence

$$\begin{aligned} \begin{aligned} d_{{\mathcal {SM}}_1, [0,T]} (U_n, W_n)&\le d_{{\mathcal {SM}}_1, [0, \tau _k / n]} (U_n, W_n) + \sup _{[\tau _k / n, T]} |U_n- W_n|\\&\le d_{{\mathcal {SM}}_1, [0,\tau _k / n]} (U_n, W_n) + \frac{1}{b_n} E \circ f^{\lfloor nT \rfloor }\rightarrow _{\mu _Z} 0 \end{aligned} \end{aligned}$$

as required. $$\square $$

#### Proof of Theorem 5.1

By Lemma 5.7, $d_{{\mathcal {SM}}_1, [0, T]}(U_n, W_n) \rightarrow _{\mu _Z} 0$ for every *T*. By Lemma 5.4, $U_n \rightarrow _{\mu _Z} W$ in ${\mathcal {SM}}_1$. Hence $W_n \rightarrow _{\mu _Z} W$ in ${\mathcal {SM}}_1$. The required convergence of $W_n \rightarrow _\mu W$ in ${\mathcal {SM}}_1$ follows from strong distributional convergence [47, Theorem 1] upon verifying that $d_{{\mathcal {SM}}_1}(W_n,W_n\circ f) \le d_{{\mathcal {SJ}}_1}(W_n,W_n\circ f) \rightarrow _\mu 0$ in the same way as [47, Corollary 3]. $\square$

### Inducing tightness in *p*-variation

In this subsection we prove Theorem 5.2. Again, we suppose without loss of generality that $f:Y\rightarrow Y$ is the tower (5.1).

#### Lemma 5.8

The family $\Vert W_n\Vert _{p\text {-}\mathrm {var}}$ is tight on $(Z, \mu _Z)$.

#### Proof

Let $\tau _n=\sum _{j=0}^{n-1}\tau \circ F^j$ and define $U_n(t) = b_n^{-1} \sum _{j=0}^{\lfloor \tau _n t \rfloor -1} v \circ f^j$ on *Z*. Note that $\Vert W_n\Vert _{p\text {-}\mathrm {var}} \le \Vert U_n\Vert _{p\text {-}\mathrm {var}}$. Let $s_i = \tau _i / \tau _n$, $i=0,\dots ,n$ and write $U_n = U'_n + U''_n$ where $U'_n|_{[s_i,s_{i+1})} \equiv U_n(s_i)$.

Observe that $U'_n$ is a time-changed version of ${\widetilde{W}}_n$ (indeed $U'_n(s_i)={\widetilde{W}}_n(i/n)$), so $\Vert U'_n\Vert _{p\text {-}\mathrm {var}} = \Vert {\widetilde{W}}_n\Vert _{p\text {-}\mathrm {var}}$. Thus the family $\Vert U'_n\Vert _{p\text {-}\mathrm {var}}$ is tight on $(Z, \mu _Z)$.

Further we bound $\int _Z \Vert U''_n\Vert _{p\text {-}\mathrm {var}} \mathrm {d}\mu _Z$. Note that $U''_n(s_i)=0$ and $\Vert 1_{[s_i,s_{i+1})}U''_n\Vert _\infty \le b_n^{-1}\Vert v\Vert _\infty \tau \circ F^i$. Hence for $t\in [s_i,s_{i+1})$, $t'\in [s_{i'},s_{i'+1})$,

$$\begin{aligned} |U_n(t)-U_n(t')|^p\le & {} \big ( b_n^{-1}\Vert v\Vert _\infty \big (\tau \circ F^i+\tau \circ F^{i'}\big )\big )^p\\\le & {} 2^{p-1}b_n^{-p}\Vert v\Vert _\infty ^p\big (\tau ^p\circ F^i+\tau ^p\circ F^{i'}\big ) . \end{aligned}$$

It follows that

$$\begin{aligned} \Vert U''_n\Vert _{p\text {-}\mathrm {var}}^p\le \sum _{i=0}^{n-1}\Vert U''_n\Vert _{p\text {-}\mathrm {var},[s_i,s_{i+1}]}^p + 2^{p}b_n^{-p}\Vert v\Vert _\infty ^p\sum _{i=0}^{n-1}\tau ^p\circ F^i . \end{aligned}$$

On $[s_i,s_{i+1}]$, there are $\tau \circ F^i-1$ jumps of size at most $b_n^{-1}\Vert v\Vert _\infty$, and one jump of size at most $b_n^{-1} \Vert v\Vert _\infty \tau \circ F^i$, so $\Vert U''_n\Vert _{p\text {-}\mathrm {var},[s_i,s_{i+1}]} \le \Vert U''_n\Vert _{1\text {-}\mathrm {var},[s_i,s_{i+1}]} \le 2b_n^{-1}\Vert v\Vert _\infty \tau \circ F^i$. Altogether, we have shown that

$$\begin{aligned} \Vert U''_n\Vert _{p\text {-}\mathrm {var}} \lesssim \Vert v\Vert _\infty b_n^{-1} \left( \sum _{j=0}^{n-1} \tau ^p \circ F^j\right) ^{1/p} . \end{aligned}$$

Now apply Proposition 4.9(c). $\square$

#### Lemma 5.9

The family $\Vert W_n\Vert _{p\text {-}\mathrm {var}}$ is tight on $(Y, \mu _Z)$ if and only if it is tight on $(Y, \mu )$.

#### Proof

Observe that $W_n(t) \circ f = W_n(t+{\textstyle \frac{1}{n}}) - b_n^{-1} v$ for all $t \ge 0$. Hence

$$\begin{aligned} \bigl | \Vert W_n\Vert _{p\text {-}\mathrm {var}} - \Vert W_n\Vert _{p\text {-}\mathrm {var}} \circ f \bigr | \le b_n^{-1} (|v| + |v| \circ f^n) \rightarrow _\mu 0 . \end{aligned}$$

Hence by Zweimüller [47, Theorem 1], $\Vert W_{n_k}\Vert _{p\text {-}\mathrm {var}}$ has the same limit in distribution (if any) on $(Y,\mu _Z)$ as on $(Y,\mu )$ for each subsequence $n_k$. The result follows. $\square$

#### Proof of Theorem 5.2

Combine Lemmas 5.8 and 5.9. $\square$

## Results for nonuniformly expanding maps

In this section, we prove results on weak convergence to a Lévy process, and tightness in *p*-variation, for a class of nonuniformly expanding maps. The weak convergence result extends work of [33] from scalar-valued observables to ${\mathbb {R}}^d$-valued observables. The result on tightness in *p*-variation is again new even for $d=1$.

We show that intermittent maps such as (1.4) and (1.5) fit our setting in Sect. 6.2.

### Nonuniformly expanding maps

Let $f:Y\rightarrow Y$ be a measurable transformation on a bounded metric space (*Y*, *d*) and let $\nu$ be a finite Borel measure on *Y*. Suppose that there exists a Borel subset $Z\subset Y$ with $\nu (Z)>0$ and an at most countable partition ${\mathcal {P}}$ of *Z* (up to a zero measure set) with $\nu (a) > 0$ for each $a \in {\mathcal {P}}$. Suppose also that there is an integrable *return time* function $\tau :Z \rightarrow \{1,2,\ldots \}$ which is constant on each $a \in {\mathcal {P}}$ with value $\tau (a)$, such that $f^{\tau (a)}(z) \in Z$ for all $z \in a$, $a \in {\mathcal {P}}$.

Define the *induced map* $F :Z \rightarrow Z$, $F(z) = f^{\tau (z)}(z)$. We assume that *f* is *nonuniformly expanding.* That is, *F* is Gibbs–Markov as in Sect. 4 and in addition there is a constant $C>0$ such that

6.1 $$\begin{aligned} d\big (f^k z, f^k z'\big ) \le C d(Fz, Fz') \qquad \text { for all }0 \le k \le \tau (a),z,z'\in a, a\in {\mathcal {P}}. \end{aligned}$$

Let $\mu _Z$ be the unique *F*-invariant probability measure absolutely continuous with respect to $\nu$. Define the ergodic *f*-invariant probability measure $\mu =\pi _*\mu _\Delta$ as in Sect. 5. Set $\bar{\tau }=\int _Z \tau \mathrm {d}\mu _Z$.

Let $v :Y \rightarrow {\mathbb {R}}^d$ be a Hölder observable with $\int _Y v \mathrm {d}\mu = 0$, and define $V,\,V^* :Z \rightarrow {\mathbb {R}}^d$ as in (5.2) and (5.3).

Let $b_n$ be a sequence of positive numbers and define $W_n$ as in (5.4). Let ${{\,\mathrm{\mathbb {P}}\,}}$ be any probability measure on *Y* that is absolutely continuous with respect to $\nu$, and regard $W_n$ as a process with paths in $D([0,1], {\mathbb {R}}^d)$, defined on the probability space $(Y, {{\,\mathrm{\mathbb {P}}\,}})$.

We can now state and prove the main results of this subsection.

#### Theorem 6.1

Suppose that:

- $V:Z\rightarrow {\mathbb {R}}^d$ is regularly varying on $(Z, \mu _Z)$ with index $\alpha \in (1,2)$ and $\sigma$ as in Definition 4.1.
- $$b_n$$ satisfies $$\lim _{n \rightarrow \infty } n \mu _Z( |V| > b_n ) = 1$$.
- $V - {{\,\mathrm{\mathbb {E}}\,}}(V \mid {\mathcal {P}}) \in L^p$ for some $p > \alpha$, where ${{\,\mathrm{\mathbb {E}}\,}}$ denotes the expectation on $(Z, \mu _Z)$.
- $$b_n^{-1} \max _{k<n} V^* \circ F^k \rightarrow _w 0$$ on $$(Z, \mu _Z)$$.

Then $W_n \rightarrow _w L_\alpha$ on $(Y,{{\,\mathrm{\mathbb {P}}\,}})$ in the ${\mathcal {SM}}_1$ topology, where $L_\alpha$ is the $\alpha$-stable Lévy process with spectral measure $\Lambda = \cos \frac{\pi \alpha }{2} \Gamma (1-\alpha ) \sigma /\bar{\tau }$.

#### Proof

Note that $|V|\le \Vert v\Vert _\infty \tau$. Let $z,z'\in a$, $a\in {\mathcal {P}}$. Then

$$\begin{aligned} |V(z)-V(z')|\le & {} \sum _{j=0}^{\tau (z)-1}|v(f^jz)-v(f^jz')| \le C_0 \sum _{j=0}^{\tau (z)-1}d(f^jz,f^jz')^\theta \\\le & {} C_0 \tau (a) d(Fz,Fz')^\theta , \end{aligned}$$

where $C_0$ is the Hölder constant for *v* and $\theta$ is the Hölder exponent, and we used condition (6.1) in the definition of nonuniformly expanding map. Hence condition (4.2) is satisfied.

Define ${\widetilde{W}}_n$ as in (5.4). By Theorem 4.2, ${\widetilde{W}}_n \rightarrow _w {\tilde{L}}_\alpha$ on $(Z, \mu _Z)$ in the ${\mathcal {SJ}}_1$ topology where ${\tilde{L}}_\alpha$ is an $\alpha$-stable Lévy process with ${\tilde{L}}_\alpha$ having spectral measure ${\tilde{\Lambda }}= \cos \frac{\pi \alpha }{2} \Gamma (1-\alpha ) \sigma$.

By Theorem 5.1, $W_n \rightarrow _w L_\alpha$ on $(Y,\mu )$ in the ${\mathcal {SM}}_1$ topology where $L_\alpha (t) = {\tilde{L}}_\alpha (t / \bar{\tau })$. This proves the result when ${{\,\mathrm{\mathbb {P}}\,}}=\mu$.

By Zweimüller [47, Theorem 1 and Corollary 3] (see also [33, Proposition 2.8]), the convergence holds not only on $(Y,\mu )$ but also on $(Y,{{\,\mathrm{\mathbb {P}}\,}})$ for any probability measure ${{\,\mathrm{\mathbb {P}}\,}}$ that is absolutely continuous with respect to $\nu$. This completes the proof. $\square$

#### Theorem 6.2

Suppose that $\tau$ is regularly varying with index $\alpha >1$ on $(Z, \mu _Z)$, and that $b_n$ satisfies $\lim _{n\rightarrow \infty } n\mu _Z(\tau >b_n)=1$. Then $\{\Vert W_n\Vert _{p\text {-}\mathrm {var}}\}$ is tight on $(Y, {{\,\mathrm{\mathbb {P}}\,}})$ for each $p>\alpha$.

#### Proof

Condition (4.2) was established in the proof of Theorem 6.1. Tightness on $(Y,\mu )$ follows from Theorems 5.2 and 4.4. Tightness on $(Y,{{\,\mathrm{\mathbb {P}}\,}})$ holds by the same argument used in the proof of Lemma 5.9. $\square$

### Intermittent maps

In this subsection, we show that Theorems 1.1 and 1.3 hold for the intermittent maps $f :[0,1] \rightarrow [0,1]$, given by (1.4) and (1.5).

We choose $Z = [\frac{1}{2},1]$ for the map (1.4), and $Z = [\frac{1}{3}, \frac{2}{3}]$ for (1.5). Let $\tau$ be the first return time to *Z*. The reference measure $\nu$ is Lebesgue and the partition ${\mathcal {P}}$ consists of maximal intervals on which the return time is constant. It is standard that the first return map $F = f^\tau$ is Gibbs–Markov, and since $f'>1$, condition (6.1) holds. Thus both maps are nonuniformly expanding.

#### Lemma 6.3

Let $v :[0,1] \rightarrow {\mathbb {R}}^d$ be Hölder with $\int v \mathrm {d}\mu = 0$ and $v(0) \ne 0$, also $v(1) \ne 0$ in case *f* is given by (1.5). Define $V,\,V^* :Z \rightarrow {\mathbb {R}}^d$ as in (5.2) and (5.3). Then

- There exists a unique absolutely continuous *f*-invariant probability measure $\mu$ on [0, 1]. Its density *h* is bounded below and is continuous on *Z*.
- *V* is regularly varying with index $\alpha$ on $(Z, \mu _Z)$. The probability measure $\sigma$ as in Definition 4.1 is given by
  $$\begin{aligned} \sigma = {\left\{ \begin{array}{ll} \delta _{v(0)/|v(0)|} &{}\quad \text { for the map (1.4)} , \\ \frac{|v(0)|^\alpha }{|v(0)|^\alpha + |v(1)|^\alpha } \delta _{v(0)/|v(0)|} + \frac{|v(1)|^\alpha }{|v(0)|^\alpha + |v(1)|^\alpha } \delta _{v(1)/|v(1)|} &{}\quad \text { for the map (1.5)} . \end{array}\right. } \end{aligned}$$
- $$\lim _{n \rightarrow \infty } n \mu _Z( |V| > b_n ) = 1$$ with $$b_n = c^{1 / \alpha } n^{1 / \alpha }$$, where
  $$\begin{aligned} c = {\left\{ \begin{array}{ll} \frac{1}{4}|v(0)|^\alpha \alpha ^\alpha h(\frac{1}{2}) \bar{\tau } &{}\quad \text { for the map (1.4)} ,\\ \frac{1}{9} \bigl ( |v(0)|^\alpha + |v(1)|^\alpha \bigr ) \alpha ^\alpha h(\frac{1}{3}) \bar{\tau } &{}\quad \text { for the map (1.5)} . \end{array}\right. } \end{aligned}$$
  Here $$\bar{\tau } = \int _Z \tau \mathrm {d}\mu _Z$$.
- $$V - {{\,\mathrm{\mathbb {E}}\,}}(V \mid {\mathcal {P}}) \in L^p$$ for some $$p > \alpha $$.
- $$n^{-1/\alpha } \max _{0\le k<n} V^* \circ F^k \rightarrow _w 0$$ on $$(Z, \mu _Z)$$.

#### Proof

We give the details for the map (1.5). The details for the map (1.4) are similar and simpler.

Let $a_1 = \frac{1}{3}$ and $a_k = a_{k+1}(1 + (3 a_{k+1})^{1/\alpha })$, $k \ge 1$. By a standard calculation, see for example [19], $a_k \sim \frac{1}{3} \alpha ^\alpha k^{-\alpha }$. Let $z_k = \frac{1}{3}(a_k + 1)$ and $z'_k = 1-z_k$. The partition ${\mathcal {P}}$ consists of the intervals $(z_{k}, z_{k-1})$ and $(z'_{k-1}, z'_{k})$, $k \ge 2$, on which $\tau$ equals *k*, and $(z_1, z'_1)$ where $\tau$ equals 1.

Observe that $F = f^\tau$ has full branches, i.e. $Fa = Z$ for every $a \in {\mathcal {P}}$, modulo zero measure. It is standard that the unique *F*-invariant absolutely continuous measure $\mu _Z$ has continuous density $h_Z$ bounded away from zero (see for example [23, Proposition 2.5]). Moreover, *h* is bounded below and $h|_Z = h_Z / \bar{\tau }$.

If $z \in (\frac{1}{3}, z_k)$ and $0 < \ell \le k$, then $f^\ell z \in (0, a_{k - \ell + 1})$, so $|f^\ell z| \lesssim (k - \ell )^{-\alpha }$. Similarly, if $z \in (z'_k, \frac{2}{3})$, then $|1-f^\ell z| \lesssim (k - \ell )^{-\alpha }$. Let $\theta \in (0,1]$ be the Hölder exponent of *v*. Without loss, we assume that $\theta < 1 / \alpha$. Define $\hat{v} = v(0) 1_{(\frac{1}{3}, \frac{1}{2})} + v(1) 1_{(\frac{1}{2},\frac{2}{3})}$ on *Z*. Then

6.2 $$\begin{aligned} \left| \ell \hat{v}(z) - \sum _{j=0}^{\ell - 1} v(f^j z) \right| \le |\hat{v}(z) - v(z)| + \sum _{j=1}^{\tau (z) - 1} |\hat{v}(z) - v(f^j z)| \lesssim \tau (z)^{\beta } \end{aligned}$$

for $\ell \le \tau (z)$, where $\beta = 1-\alpha \theta \in (0,1)$. In particular, $|\tau \hat{v} -V| \lesssim \tau ^\beta$.

By symmetry and continuity of $h_Z$,

$$\begin{aligned} {\textstyle \mu _Z\big (z>\frac{1}{2},\,\tau> k\big )= \mu _Z(z<\frac{1}{2},\,\tau > k) =\mu _Z\big (\big (\frac{1}{3}, z_k\big )\big )}\sim \frac{h_Z\big (\frac{1}{3}\big ) \alpha ^\alpha }{9 k^\alpha } . \end{aligned}$$

Let *B* be a Borel set in ${\mathbb {S}}^{d-1}$ and suppose that $v(0)/|v(0)|\in B$, $v(1)/|v(1)|\not \in B$. Then

$$\begin{aligned} \frac{\mu _Z(|\tau \hat{v}|>rt,\,\tau \hat{v}/|\tau \hat{v}|\in B)}{\mu _Z(|\tau \hat{v}|>t)}&=\frac{\mu _Z(z<\frac{1}{2},\,\tau>rt/|v(0)|)}{\mu _Z(z<\frac{1}{2},\,\tau>t/|v(0)|)+ \mu _Z(z>\frac{1}{2},\,\tau >t/|v(1)|)} \\&\rightarrow r^{-\alpha }\frac{|v(0)|^\alpha }{|v(0)|^\alpha +|v(1)|^\alpha } \quad \text {as }t\rightarrow \infty . \end{aligned}$$

The calculations for the remaining Borel sets *B* are similar, and it follows that $\tau \hat{v}$ is regularly varying with index $\alpha$ and that the probability measure $\sigma$ as in Definition 4.1 is given by the formula in part (b). By (6.2), *V* is regularly varying with index $\alpha$ and the same $\sigma$, proving part (b).

Moreover, $\mu _Z(|\tau \hat{v}|>n)\sim cn^{-\alpha }$ with *c* as in part (c), so $\mu _Z(|V|>n)\sim cn^{-\alpha }$ by (6.2). Part (c) follows by Remark 4.3(a).

It is immediate from (6.2) that $|V(z) - V(z')| \lesssim \tau (a)^\beta$ for all $z,z'\in a$, $a\in {\mathcal {P}}$. Part (d) follows by Remark 4.3(b).

Finally, it follows from (6.2) that $V^* \lesssim \tau ^\beta$, from which $V^* \in L^q (\mu _Z)$ for some $q > \alpha$, and

$$\begin{aligned} \int \Bigl ( n^{-1/\alpha } \max _{0\le k<n} V^* \circ F^k \Bigr )^q \mathrm {d}\mu _Z&\le n^{-q / \alpha } \sum _{k < n} \int (V^*)^q \circ F^k \mathrm {d}\mu _Z \\&= n^{-q / \alpha + 1} \Vert V^*\Vert _q^q \rightarrow 0 . \end{aligned}$$

This proves (e) and completes the proof of the lemma. $\square$

Theorems 1.1 and 1.3 now follow from Theorems 6.1 and 6.2. Moreover, $L_\alpha$ is identified as the $\alpha$-stable Lévy process with spectral measure $\Lambda = c \cos \frac{\pi \alpha }{2} \Gamma (1-\alpha ) \sigma /\bar{\tau }$ with *c* and $\sigma$ as in Lemma 6.3.

Finally, as a consequence of these results combined with Theorem 2.6, we can record the desired conclusion for homogenisation of fast–slow systems with fast dynamics given by one of the intermittent maps in Sect. 1.

#### Corollary 6.4

Consider the intermittent map (1.4) or (1.5) with $\alpha \in (1,2)$ and let $v:Y\rightarrow {\mathbb {R}}^d$ be Hölder with $\int _Y v\mathrm {d}\mu =0$ and $v(0)\ne 0$, also $v(1)\ne 0$ in case of (1.5).

Consider the fast–slow system (1.1) with initial condition $x^{(n)}_0 = \xi _n$ such that $\lim _{n\rightarrow \infty }\xi _n = \xi$. Suppose that $a\in C^{\beta }({\mathbb {R}}^m,{\mathbb {R}}^m)$, $b \in C^\gamma ({\mathbb {R}}^m,{\mathbb {R}}^{m\times d})$ for some $\beta >1$, $\gamma > \alpha$. Define $W_n$ as in (1.2) and $X_n(t) = x_{\lfloor nt \rfloor }^{(n)}$. Let ${{\,\mathrm{\mathbb {P}}\,}}$ be any probability measure on *Y* that is absolutely continuous with respect to Lebesgue, and regard $W_n$ and $X_n$ as processes on $(Y,{{\,\mathrm{\mathbb {P}}\,}})$.

Let $\ell _{k}$ denote the linear path function on ${\mathbb {R}}^k$ and let $\phi _{b}$ be the path function on ${\mathbb {R}}^{d+m}$ as in Definition 2.5. Fix $p>\alpha$. Then

$$\begin{aligned} ((W_n, X_n), \ell _{d+m}) \rightarrow _w ((L_\alpha , X), \phi _{b}) \qquad \text {as} \qquad n \rightarrow \infty \end{aligned}$$

in $({\mathscr {D}}^{p\text {-}\mathrm {var}}([0,1],{\mathbb {R}}^{d +m}), {\varvec{\alpha }}_{p\text {-}\mathrm {var}})$, where $L_\alpha$ is the $\alpha$-stable Lévy process with spectral measure $\Lambda = c \cos \frac{\pi \alpha }{2} \Gamma (1-\alpha ) \sigma /\bar{\tau }$ with *c* and $\sigma$ as in Lemma 6.3, and *X* is the solution of the Marcus differential equation (2.4). $\square$

## Footnotes

1 Generated from https://khu.dedyn.io/work/scaled-graphs/fast-slow/.

## References

[1] Aaronson J, Denker M (2001). Local limit theorems for partial sums of stationary sequences generated by Gibbs–Markov maps. Stoch. Dyn..

[2] Applebaum D (2009). Lévy Processes and Stochastic Calculus.

[3] Bingham NH, Goldie CM, Teugels JL (1987). Regular Variation.

[4] Bradley RC (2005). Basic properties of strong mixing conditions a survey and some open questions. Probab. Surv..

[5] Chechkin A, Pavlyukevich I (2014). Marcus versus Stratonovich for systems with jump noise. J. Phys. A.

[6] Chevyrev I (2018). Random walks and Lévy processes as rough paths. Probab. Theory Relat. Fields.

[7] Chevyrev I, Friz PK (2019). Canonical RDEs and general semimartingales as rough paths. Ann. Probab..

[8] Chevyrev, I., Friz, P.K., Korepanov, A., Melbourne, I., Zhang, H.: Multiscale systems, homogenization, and rough paths. In: Friz, P., et al. (eds.) Probability and Analysis in Interacting Physical Systems: In Honor of S.R.S. Varadhan, Berlin, August, 2016 (.), Springer Proceedings in Mathematics and Statistics, vol. 283, pp. 17–42 (2019)

[9] Chevyrev, I., Friz, P.K., Korepanov, A., Melbourne, I., Zhang, H.: Deterministic homogenization under optimal moment assumptions for fast–slow systems. Part 2. Preprint (2019)

[10] Dolgopyat D (2004). Limit theorems for partially hyperbolic systems. Trans. Am. Math. Soc..

[11] Dolgopyat D (2005). Averaging and invariant measures. Mosc. Math. J..

[12] Friz P, Hairer M (2014). A Course on Rough Paths. With an Introduction to Regularity Structures.

[13] Friz PK, Shekhar A (2017). General rough integration, Lévy rough paths and a Lévy–Kintchine-type formula. Ann. Probab..

[14] Friz PK, Victoir NB (2010). Multidimensional Stochastic Processes as Rough Paths.

[15] Friz PK, Zhang H (2018). Differential equations driven by rough paths with jumps. J. Differ. Equ..

[16] Gottwald G, Melbourne I (2013). Homogenization for deterministic maps and multiplicative noise. Proc. R. Soc. Lond. A.

[17] Gouëzel S (2004). Central limit theorem and stable laws for intermittent maps. Probab. Theory Relat. Fields.

[18] Gouëzel S (2007). Statistical properties of a skew product with a curve of neutral points. Ergod. Theory Dyn. Syst..

[19] Holland M (2005). Slowly mixing systems and intermittency maps. Ergod. Theory Dyn. Syst..

[20] Kelly D, Melbourne I (2016). Smooth approximation of stochastic differential equations. Ann. Probab..

[21] Kelly D, Melbourne I (2017). Homogenization for deterministic fast–slow systems with multidimensional multiplicative noise. J. Funct. Anal..

[22] Kocheim, D., Pühringer, F., Zweimüller, R.: A functional stable limit theorem for Gibbs–Markov maps. Preprint (2018)

[23] Korepanov A, Kosloff Z, Melbourne I (2019). Explicit coupling argument for nonuniformly hyperbolic transformations. Proc. Edinb. Math. Soc..

[24] Korepanov, A., Kosloff, Z., Melbourne, I.: Deterministic homogenization under optimal moment assumptions for fast–slow systems. Part 1. Preprint (2020)

[25] Kurtz TG, Pardoux E, Protter P (1995). Stratonovich stochastic differential equations driven by general semimartingales. Ann. Inst. H. Poincaré Probab. Stat..

[26] Lépingle D (1976). La variation d’ordre $$p$$ des semi-martingales. Z. Wahrscheinlichkeitstheorie und Verw. Gebiete.

[27] Liverani C, Saussol B, Vaienti S (1999). A probabilistic approach to intermittency. Ergod. Theory Dyn. Syst..

[28] Lyons T (1994). Differential equations driven by rough signals. I. An extension of an inequality of L.C. Young. Math. Res. Lett..

[29] Marcus, S.I.: Modeling and approximation of stochastic differential equations driven by semimartingales. Stochastics **4**, 223–245 (1980/81)

[30] Melbourne I, Nicol M (2005). Almost sure invariance principle for nonuniformly hyperbolic systems. Commun. Math. Phys..

[31] Melbourne I, Török A (2004). Statistical limit theorems for suspension flows. Isr. J. Math..

[32] Melbourne I, Stuart A (2011). A note on diffusion limits of chaotic skew product flows. Nonlinearity.

[33] Melbourne I, Zweimüller R (2015). Weak convergence to stable Lévy processes for nonuniformly hyperbolic dynamical systems. Ann Inst. H. Poincaré (B) Probab. Stat..

[34] Pavliotis GA, Stuart AM (2008). Multiscale Methods.

[35] Pène F (2002). Averaging method for differential equations perturbed by dynamical systems. ESAIM Probab. Stat..

[36] Pisier G, Xu QH (1988). The strong $$p$$-variation of martingales and orthogonal series. Probab. Theory Relat. Fields.

[37] Pomeau Y, Manneville P (1980). Intermittent transition to turbulence in dissipative dynamical systems. Commun. Math. Phys..

[38] Ratner M (1973). The central limit theorem for geodesic flows on $$n$$-dimensional manifolds of negative curvature. Isr. J. Math..

[39] Samorodnitsky G, Taqqu M (1994). Stable non-Gaussian random processes: stochastic models with infinite variance.

[40] Skorohod AV (1956). Limit theorems for stochastic processes. Theory Probab. Appl..

[41] Tyran-Kamińska M (2010). Convergence to Lévy stable processes under some weak dependence conditions. Stochastic Process. Appl..

[42] Tyran-Kamińska M (2010). Weak convergence to Lévy stable processes in dynamical systems. Stoch. Dyn..

[43] Whitt W (2002). Stochastic-process limits.

[44] Williams D (2001). Path-wise solutions of stochastic differential equations driven by Lévy processes. Rev. Mat. Iberoam.

[45] Wong E, Zakai M (1965). On the convergence of ordinary integrals to stochastic integrals. Ann. Math. Statist..

[46] Zweimüller R (2003). Stable limits for probability preserving maps with indifferent fixed points. Stoch. Dyn..

[47] Zweimüller R (2007). Mixing limit theorems for ergodic transformations. J. Theoret. Probab..

